# Exosomal lncRNA DOCK9-AS2 derived from cancer stem cell-like cells activated Wnt/β-catenin pathway to aggravate stemness, proliferation, migration, and invasion in papillary thyroid carcinoma

**DOI:** 10.1038/s41419-020-02827-w

**Published:** 2020-09-11

**Authors:** Wencheng Dai, Xiaoxia Jin, Liang Han, Haijing Huang, Zhenhua Ji, Xinjiang Xu, Mingming Tang, Bin Jiang, Weixian Chen

**Affiliations:** 1 Department of Head and Neck Surgery, Nantong Tumor Hospital, Affiliated Tumor Hospital of Nantong University, No.30 North Tongyang Road, Pingchao, 226361 Nantong, Jiangsu China; 2 Department of Pathology, Nantong Tumor Hospital, Affiliated Tumor Hospital of Nantong University, No.30 North Tongyang Road, Pingchao, 226361 Nantong, Jiangsu China; 3 Laboratory of Nantong Third People’s Hospital, No.60 Youth Middle Road, Chongchuan District, 226001 Nantong, Jiangsu China

**Keywords:** Head and neck cancer, Head and neck cancer

## Abstract

Exosomal long non-coding RNAs (lncRNAs) are crucial factors that mediate the extracellular communication in tumor microenvironment. DOCK9 antisense RNA2 (DOCK9-AS2) is an exosomal lncRNA which has not been investigated in papillary thyroid carcinoma (PTC). Based on the result of differentially expressed lncRNAs in PTC via bioinformatics databases, we discovered that DOCK9-AS2 was upregulated in PTC, and presented elevation in plasma exosomes of PTC patients. Functionally, DOCK9-AS2 knockdown reduced proliferation, migration, invasion, epithelial-to-mesenchymal (EMT) and stemness in PTC cells. PTC-CSCs transmitted exosomal DOCK9-AS2 to improve stemness of PTC cells. Mechanistically, DOCK9-AS2 interacted with SP1 to induce catenin beta 1 (CTNNB1) transcription and sponged microRNA-1972 (miR-1972) to upregulate CTNNB1, thereby activating Wnt/β-catenin pathway in PTC cells. In conclusion, PTC-CSCs-derived exosomal lncRNA DOCK9-AS2 activated Wnt/β-catenin pathway to aggravate PTC progression, indicating that DOCK9-AS2 was a potential target for therapies in PTC.

## Introduction

Papillary thyroid cancer (PTC) takes up around 80% of thyroid cancer (TC) cases^1^. Treatment outcome of PTC is generally satisfactory, and with appropriate treatment, over 95% of PTC patients can survive longer than 5 years^2^. However, there are still approximately 15% of PTC cases presenting aggressive behavior and unsatisfactory prognosis^3^. Therefore, more efforts are required for the improvement of targeted therapy and diagnosis in PTC.

Long non-coding RNAs (lncRNAs) are known as transcripts without protein-coding ability and consist over 200 nucleotides^4^. LncRNAs can modulte gene expression at diverse levels, such as transcriptional level^5,6^, and post-transcriptional level^7^. Also, lncRNAs have been linked to cancer-related behaviors such as proliferation^8^, stemness^9,10^, and metastasis^11,12^. Mounting lncRNAs have been illustrated to participate in PTC, such as PTCSC2 and n384546^13,14^. DOCK9 antisense RNA2 (DOCK9-AS2) is identified as an important lncRNA related to atherosclerosis^15^. Through GEPIA and circlncRNAnet, DOCK9-AS2 is upregulated in thyroid carcinoma (THCA) specimens, indicating that DOCK9-AS2 participates in PTC development. However, no report has demonstrated the function and modulatory mechanism of DOCK9-AS2 in PTC yet.

Cancer stem-like cells (CSCs) are a small population of tumor cells that contribute to tumor initiation, metastasis and therapy-resistance^16,17^. The equilibrium state between CSCs and non-CSCs is highly dynamic^18,19^, which means that under certain circumstances, non-CSCs could differentiate into CSCs and CSCs into non-CSCs^20^. The involvement of lncRNAs in this process are increasingly documented, even in PTC. For instance, LINC00311 strengthens PTC cell stemness via miR-330-5p/TLR4 pathway^21^. However, the association of DOCK9-AS2 with PTC-CSCs has not been established.

Exosomes are small (30–150 nm) membranous vesicles originated from the multi-vesicular endosomes^22^,which can transfer some intracellular cargoes between cells^23,24^. Also, tumor cells release exosomes to regulate the tumor microenvironment and impact various target cells^25,26^. Studies have revealed that the molecular cross-talk between CSCs and non-CSCs is important for the CSCs-non-CSCs dynamic equilibrium^20^. Moreover, numerous exosomal lncRNAs are reported to transmit signals and phenotypes between cancer cells, so are in PTC cells^27^. However, whether DOCK9-AS2 functions as an exosomal lncRNA related to PTC-CSCs in PTC microenvironment is unclear.

Therefore, this study proposed to explore whether and how DOCK9-AS2 mediated the crosstalk between CSCs and naïve PTC cells via exosome transmission in PTC.

## Materials and methods

### Patient and tissues collection

Fifty four pairs of PTC and adjacent normal tissue samples of patients with PTC were collected at Nantong Tumor Hospital, with the written informed consents from all participants and the approval from the Ethics Committee of Nantong Tumor Hospital. Patients treated with radiotherapy or chemotherapy before surgery were excluded. After surgical resection, all tissue samples were instantly frozen in liquid nitrogen and then stored at −80 °C.

### Cell culture

Human thyroid epithelial cell Nthy-ori3-1 and PTC cells (BCPAP, TPC1), all from the American Type Culture Collection (ATCC; Manassas, VA, USA), were cultured in DMEM (Gibco, Grand Island, NY, USA) with 10% fetal bovine serum (FBS; Gibco) and 1% penicillin/streptomycin at 37 °C with 5% CO_2_. Cells were cultivated in serum-free stem cell medium (SCM) which was prepared using DMEM/F12 (Gibco), 10 ng/ml bFGF (PeproTech, London, UK), 10 ng/ml EGF (PeproTech) and N2 supplements (Gibco) for 14 days to enrich PTC-CSCs as cancer spheroids, followed by dissociation with TryPLE Express (Gibco).

### Real-time quantitative PCR (RT-qPCR)

Using Trizol reagent (Invitrogen, Grand Island, NY, USA), the extracted RNA samples were and processed with the PrimeScript™ II 1st Strand cDNA Synthesis Kit (Takara Bio, Otsu, Japan) to generate the first-strand cDNA. RT-qPCR was run with ABI 7900 system (Applied biosystems, Foster City, CA, USA) and SYBR Green assays (TaKaRa). With GAPDH or U6 as the endogenous control, gene expression was determined via 2^−ΔΔCt^ method.

### Exosome isolation

Exosomes were isolated from plasma or culture medium as previously suggested^28^. In short, samples after centrifugation were subjected to Exoquick exosome precipitation solution (System Biosciences) to obtain exosomes.

### Transmission electron microscopy (TEM) and nanoparticle tracking analysis (NTA)

To conduct TEM for exosomes morphology, the fixed exosomes in 2% paraformaldehyde (Sigma-Aldrich, St. Louis, MO, USA) were moved onto the carbon/Formvar-coated grid (Ted Pella, Inc., Tustin, CA, USA) at room temperature for 30 min. After washing in PBS, the grid was cultured with 1% glutaraldehyde (Sigma-Aldrich), followed by the observation under JEOL JEM-2000EXII microscope (Jeol Ltd, Tokyo, Japan). To perform NTA for size distribution, exosomes were diluted to 10^6^ to 10^9^ particles/ml, then analyzed by NanoSight instrument (Malvern Panalytical, Malvern, UK).

### Western blotting

Cell protein samples were obtained and separated via electrophoresis on 10% SDS polyacrylamide gels, and transferred onto PVDF membranes. The primary antibodies including anti-CD63 (ab217345), anti-CD81 (ab79559), anti-TSG-101 (ab125011), anti-Alix (ab186429), anti-Calnexin (ab92573), anti-E-cadherin (ab1416), anti-N-cadherin (ab18203), anti-MMP2 (ab97779), anti-MMP7 (ab5706), anti-CD133 (ab19898), anti-Nanog (ab109250), anti-OCT4 (ab18976), anti-SOX2 (ab97959), anti-EpCAM (ab223582), anti-ALDH1A1 (ab52492), anti-p-GSK-3β (ab75745), anti-GSK-3β (ab93926), anti-β-catenin (ab16051), anti-c-Myc (ab32072), anti-H3 (ab1791), anti-SP1 (ab227383), and anti-GAPDH (ab181602), as well as secondary antibody were all acquired from Abcam (Cambridge, MA, USA). The enhanced chemiluminescence reagent (Santa Cruz Biotechnology, Santa Cruz, CA, USA) was applied for detecting protein bands.

### Fluorescence in situ hybridization (FISH)

TPC1 and BCPAP cells were 80% confluent at the time of fixation and were then pre-hybridized with 1× PBS/0.5% Triton X-100. Following hybridization with Cy5-labeled probes specific to DOCK9-AS2 all night, the nuclei were counterstained with DAPI solution from Beyotime (Guangzhou, China). Images were finally taken by confocal microscope (Leica Microsystems, Mannheim, Germany).

### Subcellular fractionation

RNAs from nucleus and cytoplasm of PTC cells were isolated with PARIS™ Kit (Ambion, Austin, TX, USA) obeying supplier’s recommendation. The reaped PTC cells underwent re-suspension in the cell fraction buffer. Thereafter, cells underwent 10-min culturing on ice. The upper solution was then removed by centrifugation, the upper solution was removed and the nuclear pellet was obtained and kept for RNA isolation applying cell disruption buffer. Finally, the isolated RNAs underwent RT-qPCR detection, normalizing to cytoplasm (GAPDH) and nuclear (U6) controls.

### Transfection

Transfection of plasmids in TPC1 or BCPAP cells was carried out for 48 h in line with the protocol of Lipofectamine 2000 (Invitrogen). The DOCK9-AS2 or SP1-specific shRNAs (sh-DOCK9-AS2#1/2, sh-SP1), and the pcDNA3.1/SP1, pcDNA3.1/CTNNB1, as well as the negative controls (sh-NC and empty pcDNA3.1 vector), were all synthesized at GenePharma (Shanghai, China). MiR-1972 mimic and miR-1972 inhibitor (GenePharma) were applied for the overexpression and silencing of miR-1972, with NC mimic and NC inhibitor as controls.

### Cell counting kit-8 (CCK-8)

PTC cells were harvested and put into 96-well plates (1000 cells/well), following addition of 10 μl of CCK8 reagent from DOJINDO (Tokyo, Japan) for 2 h at 37 °C. At last, the absorption at 450 nm was recorded using microplate reader (Tecan, Männedorf, Switzerland).

### Colony formation

Eight hundred PTC cells were plated per well in 6-well plates with complete culture medium for 14 days. The fixed cells in 4% of paraformaldehyde (Sigma-Aldrich) were treated with crystal violet staining (Sigma-Aldrich) for counting.

### Invasion assay

Invasive ability of PTC cells were analyzed by Matrigel-coated transwell chamber (Corning Incorporated, Corning, NY, USA). 10,000 cells in serum-free medium were placed to the upper chamber. Complete medium was added into the lower chamber. Invading cells were subjected to fixing in paraformaldehyde and stained in crystal violet solution, followed by counting with microscope (magnification, ×100).

### Wound-healing assay

PTC cells were cultivated in 6-well plates all night, cell layer was scratched using sterile plastic pipette tips for creating wounds. After 24 h of incubation with medium, images were obtained by microscopy (Olympus, Tokyo, Japan) at ×100 magnification.

### Immunofluorescence staining (IF)

PTC cells on cover slips were fixed with cold acetone for 20 min and treated with primary antibodies against E-cadherin or N-cadherin all night, followed by culturing with secondary antibody for 1 h. DAPI was used to dye cell nuclei. After fluorescent staining, cells were visualized with fluorescence microscope (Olympus).

### Sphere-forming assay

PTC cells were initially placed into 96-well ultralow attachment plates (Corning) with 10 cells per well in sphere medium for 7 days. The cell cluster with the diameter more than 50 mm was defined as sphere cells and counted. Sphere formation efficiency equaled to number of spheres/number of seeded cells, and control group was set as 1.

### Flow cytometry analysis and cell sorting

The blocking solution containing 2 mM of EDTA, 1% bovine serum albumin (BSA) and PBS was utilized for preparing the single-cell suspension. Then, samples were hybridized with fluorescence conjugated primary antibodies including anti-CD44-APC (BioLegend, San Diego, CA, USA) and anti-CD133-PE (Miltenyi Biotech, Bergish-Gladbach, Germany) Alexa Fluor-488 (Invitrogen). Cell debris was removed by scatter signals. After the samples were stained with the indicated fluorescence-labeled antibodies, the fluorescence compensation was adjusted. Cells under more than 80% purity upon sorting were collected for followed analysis. Data acquisition was performed by FACS Calibur flow cytometer (Becton-Dickinson, Sparks, MD, USA) and cell sorting was performed with FACSAria system (Becton-Dickinson), followed by CellQuest (Becton-Dickinson) and FlowJo software analysis.

### Side-population (SP) cell detection and sorting

For detecting SP cells, cells underwent incubation with transport blockers verapamil (100 μM; Sigma-Aldrich, MO, USA), and 20 min later, cells were subjected to Hoechst 33342 incubation. Then, propidium iodide (PI, 2 μg/ml; Sigma-Aldrich, MO, USA) was applied to exclude the dead cells. Cells were analyzed by the FACSAriaII (BD Biosciences, CA, USA), and SP cells were visualized following the UV excitation based on blue emission with a 424/44 filter and red emission with a 630/22 filter (Omega Optical, Brattleboro, VT). The side and main population (MP) was separately sorted and collected from living cells (PI negative cells).

### Exosome labeling

Exosomes isolated from 1.5 × 10^6^ PTC-CSCs were cultured with 1 ml of mixed PKH67 (Sigma-Aldrich) in 100 μl of PBS for 5 min at room temperature. Exosome labeling was terminated by adding 2 ml of 0.5% BSA. Stained exosomes were obtained by Exoquick exosome precipitation solution. Exosomes in 9.6 ml of basal medium were incubated with cell sub-confluent layer for 3 h at 37 °C, and then washed and fixed. After DAPI staining for nuclei, fluorescence microscope (Zeiss Meditec AG, Jena, Germany) was applied for observation.

### Dual-luciferase reporter analysis

For Wnt/β-catenin signaling assay, TOP/FOP-Flash reporters were bought from Addgene (Cambridge, MA, USA) and co-transfected for 48 h with the gene silencing plasmids for DOCK9-AS1 (sh-DOCK9-AS1#1/2 and sh-NC) into TPC1 or BCPAP cells. For CTNNB1 promoter analysis, PTC cells were co-cultured with the pGL3-vector (Promega, Madison, WI, USA) that contained CTNNB1 promoter and specific shRNAs to DOCK9-AS1 or SP1. The wild-type (WT) or mutant (Mut) miR-1972 binding sites to DOCK9-AS2 or 3’-UTR of CTNNB1 were separately inserted into pmirGLO Dual-Luciferase miRNA Target Expression Vector (Promega), then co-transfected with miR-1972 mimic and NC mimic into cells. Reporter gene assay was performed after transfection using Dual Luciferase Assay System (Promega).

### Pull-down assay

The experiment was conducted in line with the protocol of Pierce Magnetic RNA-Protein Pull-Down Kit (Thermo Fisher Scientific, Waltham, MA, USA). Protein extracts from PTC cells were treated with biotin-labeled RNA probes and streptavidin agarose magnetic beads from Thermo Fisher Scientific. The retrieved RNA and protein levels were measured by RT-qPCR and western blotting.

### Chromatin immunoprecipitation (ChIP)

According to the instruction of Magna ChIP Kit (Millipore), ChIP assay was performed. After cross-linking, chromatin was broken to 200–500-bp fragments and immunoprecipitated with anti-SP1 or anti-IgG antibody as control (Millipore) at 4 °C overnight. Following the addition of magnetic beads, the precipitated chromatin was purified for RT-qPCR.

### RNA immunoprecipitation (RIP)

In accordance with the user guide of Magna RIP™ Kit (Millipore), RIP assay was carried out. 1 × 10^7^ PTC cells were reaped for culturing in RIP lysis buffer. Thereafter, the lysates were immunoprecipitated with anti-Ago2 or anti-IgG antibody (Millipore). The level of recovered RNA was acquired via RT-qPCR.

### Tumor xenograft formation in nude mice assay

Twenty male BALB/c nude mice of 6-weeks were purchased from the National Laboratory Animal Center (Beijing, China) and preserved in the SPF-grade animal laboratory, with the approval of the Animal Research Ethics Committee of Nantong Tumor Hospital. The exosomes isolated from TPC1-CSCs transfected with sh-NC or sh-DOCK9-AS2#1 were incubated with TPC1 cells, with PBS as control. Nude mice were randomly divided into 4 groups (5 mice each group) and injected subcutaneously with the cultured TPC1 cells for 28 days, with tumor volume recorded every 4 days. After sacrificing mice, tumors were carefully excised and weighed. To construct in vivo metastasis model, mice were injected intravenously with TPC1 cells with indicated treatment from the tail vein. Six weeks later, lungs of all mice were collected, with metastatic nodules secondary to lung counted manually and observed by hematoxylin and eosin (HE) staining.

### Immunohistochemistry analysis (IHC)

Xenograft tumor tissue samples were fixed by formalin and dehydrated, the 4-mm-thick sections were cut from paraffin-embedded samples. Consecutive sections were analyzed by IHC using antibodies against Ki67, PCNA, E-cadherin, N-cadherin (Abcam). After treatment with secondary antibody, sections were subjected to microscope (Olympus).

### Hematoxylin and eosin (HE) staining

To assess tumor metastasis, paraffin-embedded sections were stained by hematoxylin for 5 min. After washing in flowing water and dehydration, sections were stained with eosin and observed.

### Statistical analyses

Data of all experiments with independent triplication were analyzed by Prism 6 software (GraphPad, La Jolla, CA, USA) and expressed as mean ± Standard Deviation (S.D.). Correlation analysis was assayed by Pearson’s *χ*^2^ analysis. Group comparisons were analyzed with Student’s *t*-test and one-way ANOVA, and the difference was significant with a value of *p* < 0.05.

## Results

### DOCK9-AS2 was upregulated in PTC and was enriched in the exosomes from PTC plasma and cells

**Fig. 1:**
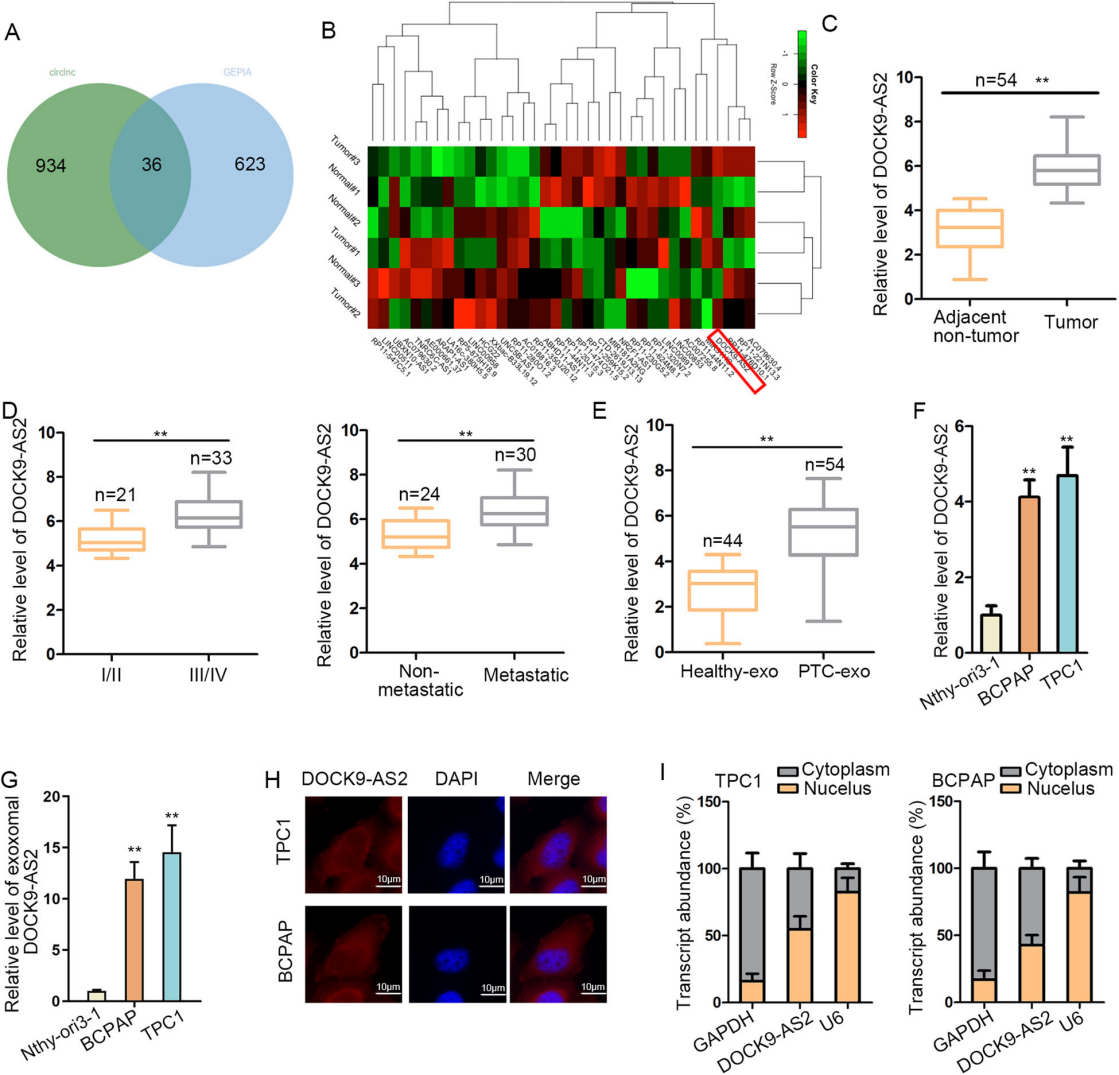
DOCK9-AS2 was upregulated in PTC and was highly expressed in exosomes in PTC samples. **a** Venn diagram showed 36 lncRNAs with significant upregulation in PTC samples in the intersection of predicted results from circlncRNAnet and GEPIA2. **b** 36 candidate lncRNAs were analyzed in PTC tissues (*n* = 3) by RT-qPCR, versus the matched para-tumorous tissues (*n* = 3). **c** RT-qPCR of DOCK9-AS2 level in PTC tumor samples (*n* = 54) versus paired para-tumorous samples (*n* = 54). **d** RT-qPCR data of DOCK9-AS2 level in PTC patients at stage III/IV (*n* = 33) versus stage I/II (*n* = 21) and in metastatic patients (*n* = 30) versus non-metastatic patients (*n* = 24). **e** RT-qPCR analysis for the DOCK9-AS2 level in the plasma exosomes from PTC cases (*n* = 54) healthy control (*n* = 44). **f**. RT-qPCR of DOCK9-AS2 expression in PTC cell lines (TPC1 and BAPAB) compared with normal cell line (Nthy-ori3-1). **g** RT-qPCR detection of DOCK9-AS2 level in exosomes from PTC cells versus normal Nthy-ori3-1 cells. **h** FISH images of DOCK9-AS2 in PTC cells. Scale Bar: 10 μm. **i** Subcellular fractionation for DOCK9-AS2 expression in cytoplasm and nucleus of PTC cells. Scale bar: 10 μm. GAPDH and U6 were cytoplasmic and nuclear references. ^**^P < 0.01. Error bar denotes Mean ± S.D.

First, to identify differentially expressed lncRNAs in PTC, we browsed circlncRNAnet (log2 fold change > 1, *P* < 0.05) (http://120.126.1.61/circlnc/circlncRNAnet/lncRNA_TCGA/index.php) and GEPIA2 (log2 fold change >1, *P* < 0.05) (http://gepia2.cancer-pku.cn/#index) and found 36 lncRNAs exhibiting upregulation in PTC samples (Fig. 1a). To narrow the selection, we tested the 36 candidates in three pairs of clinical PTC specimens and recognized DOCK9-AS2 as the most upregulated lncRNA in PTC tissues versus matched non-tumor tissues (Fig. 1b). Furthermore, GEPIA database showed high DOCK9-AS2 level in THCA specimens (*n* = 512) versus normal specimens (*n* = 337) (Fig. S1A). These results indicated that DOCK9-AS2 was potentially linked to PTC. Then, we confirmed DOCK9-AS2 upregulation in 54 PTC tumor samples versus paired non-tumor ones (Fig. 1c). Further, we observed higher DOCK9-AS2 level in PTC patients at advanced stage and those with metastasis (Fig. 1d). These data indicated that DOCK9-AS2 might participate in PTC tumor growth and metastasis.

Evidence supported that lncRNAs are deeply involved in the communication of tumor microenvironment^28,29^. Therefore, we wondered whether DOCK9-AS2 existed in exosomes. We extracted the exosomes from plasma of 54 PTC patients and 44 healthy controls. Besides, we revealed that these exosomes all had a cup-shaped morphology and their size distributed from 50 to 130 nm, with the existence of several exosomal markers (including CD81, CD63, TSG-101, and Alix) but without Calnexin (an endoplasmic reticulum specific protein) (Fig. S1B). RT-qPCR analysis revealed that DOCK9-AS2 was upregulated in the plasma exosomes from PTC cases versus healthy control (Fig. 1e). In addition, DOCK9-AS2 level was more than 4 fold higher in PTC cell lines (TPC1 and BAPAB) than in Nthy-ori3-1 cell line (Fig. 1f). Also, exosomes derived from TPC1, BAPCB, and Nthy-ori3-1 cells were confirmed according to the size distribution (50–120 nm), the cup-shaped characteristics, and the presence of exosomal markers (Fig. S1C). Interestingly, we found that DOCK9-AS2 was 3–4 fold higher in TPC1 and BCPAP-derived exosomes than in TPC1 and BCPAP cells, but DOCK9-AS2 level presented no significant difference between normal Nthy-ori3-1 cells-derived exosomes and Nthy-ori3-1 cells (Fig. S1D). Consistently, we demonstrated that DOCK9-AS2 level in exosomes from TPC1 and BCPAP was about 10 fold change compared to that in exosomes from Nthy-ori3-1 cells (Fig. 1g). Thus, we guessed that exosomal DOCK9-AS2 might be important in PTC.

Subsequently, we investigated the features of DOCK9-AS2. Coding Potential Calculator (CPC) tool (http://cpc.cbi.pku.edu.cn/programs/cpc.do) confirmed the low coding potential score of DOCK9-AS2 (about −1.25), indicating DOCK9-AS2 as a bona fide non-coding RNA (Fig. S1E). Moreover, FISH staining and subcellular fractionation identified the distribution of DOCK9-AS2 in both cytoplasm and nucleus of PTC cells (Fig. 1h–i). Together, DOCK9-AS2 upregulated in PTC was enriched in the exosomes.

### DOCK9-AS2 silence abrogated proliferation, migration, invasion, EMT, and stemness of PTC cells

**Fig. 2:**
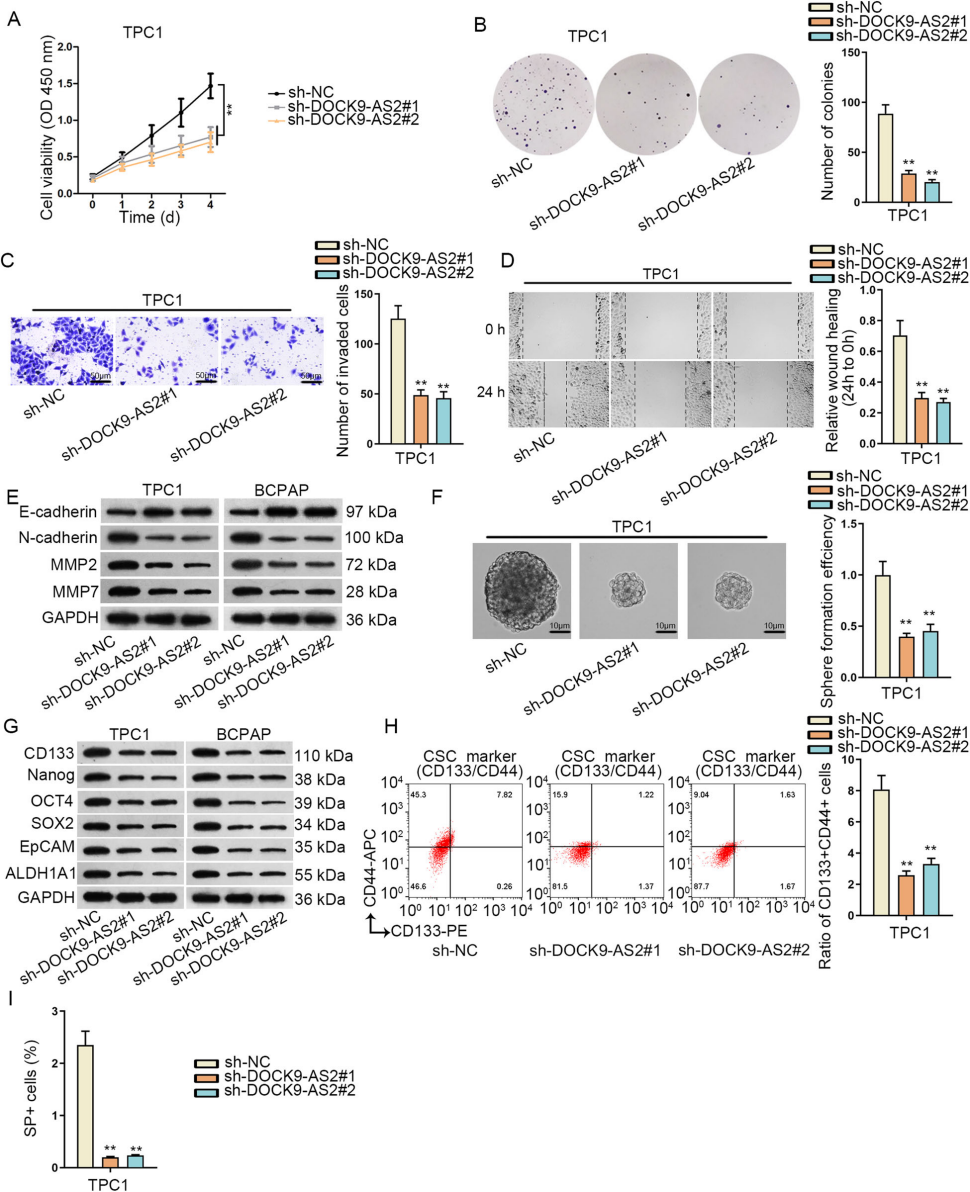
DOCK9-AS2 silence repressed proliferation, migration, invasion, EMT, and stemness of PTC cells. **a** Viable PTC cells were detected by CCK-8 under DOCK9-AS2 knockdown. **b** Images of colonies from PTC cells after transfection with sh-DOCK9-AS2#1/2 was taken and colony number was counted manually. **c** Images of invasive PTC cells under DOCK9-AS2 deficiency in transwell assay was captured and cell number per field was evaluated. Scale bar: 100 μm. **d** Pictures of scratch wound of PTC cells at 0 and 24 h under DOCK9-AS2 knockdown was taken and wound width was assessed. **e** Western blots of E-cadherin, N-cadherin, MMP2, and MMP7 in PTC cells with DOCK9-AS2 knockdown. **f** Images of 3rd generation spheres formed by PTC cells under DOCK9-AS2. Scale bar: 100 μm. Sphere formation efficiency equaled to sphere number/number of seeded cells, and control group was set as 1. **g** The levels of stem specific genes including CD133, Nanog, OCT4, SOX2, EpCAM, and ALDH1A1 were analyzed by western blotting. **h** Flow cytometry plot of CD44 + CD133 + TPC1 cells and quantification of CD44 + CD133 + ratio. **i** The SP cells were sorted from TPC1 cells with DOCK9-AS2 knockdown by flow cytometry. ^**^P < 0.01. Error bar denotes Mean ± S.D.

Since we suggested the potent correlation of DOCK9-AS2 with tumor growth and metastasis in PTC, we detected whether DOCK9-AS2 affected growth and metastasis-related behaviors in PTC cells via performing loss-of-function assays. As a result, we uncovered that silencing DOCK9-AS2 led to reduced viability and proliferation in TPC1 and BCPAP cells (Fig. 2a, b and S2A–C). Likewise, the invasion and migration of these two cells were also hindered after DOCK9-AS2 suppression (Fig. 2c–e and S2D–F). Also, PTC cells with DOCK9-AS2 knockdown presented restrained tumor sphere forming efficiency and lowered levels of stemness-related proteins including CD133, Nanog, OCT4, SOX2, EpCAM, and ALDH1A1 (Fig. 2f, g and S2G). Besides, ratio of CD44^+^CD133^+^ PTC cells, and that of mature ones with side population (SP) phenotype (SP^+^ PTC), were both lessened upon DOCK9-AS2 knockdown (Fig. 2h–i and S2H–I). Jointly, DOCK9-AS2 knockdown impaired malignant phenotypes of PTC cells.

### Exosomal DOCK9-AS2 derived from PTC-CSCs improved malignant behaviors in PTC

**Fig. 3:**
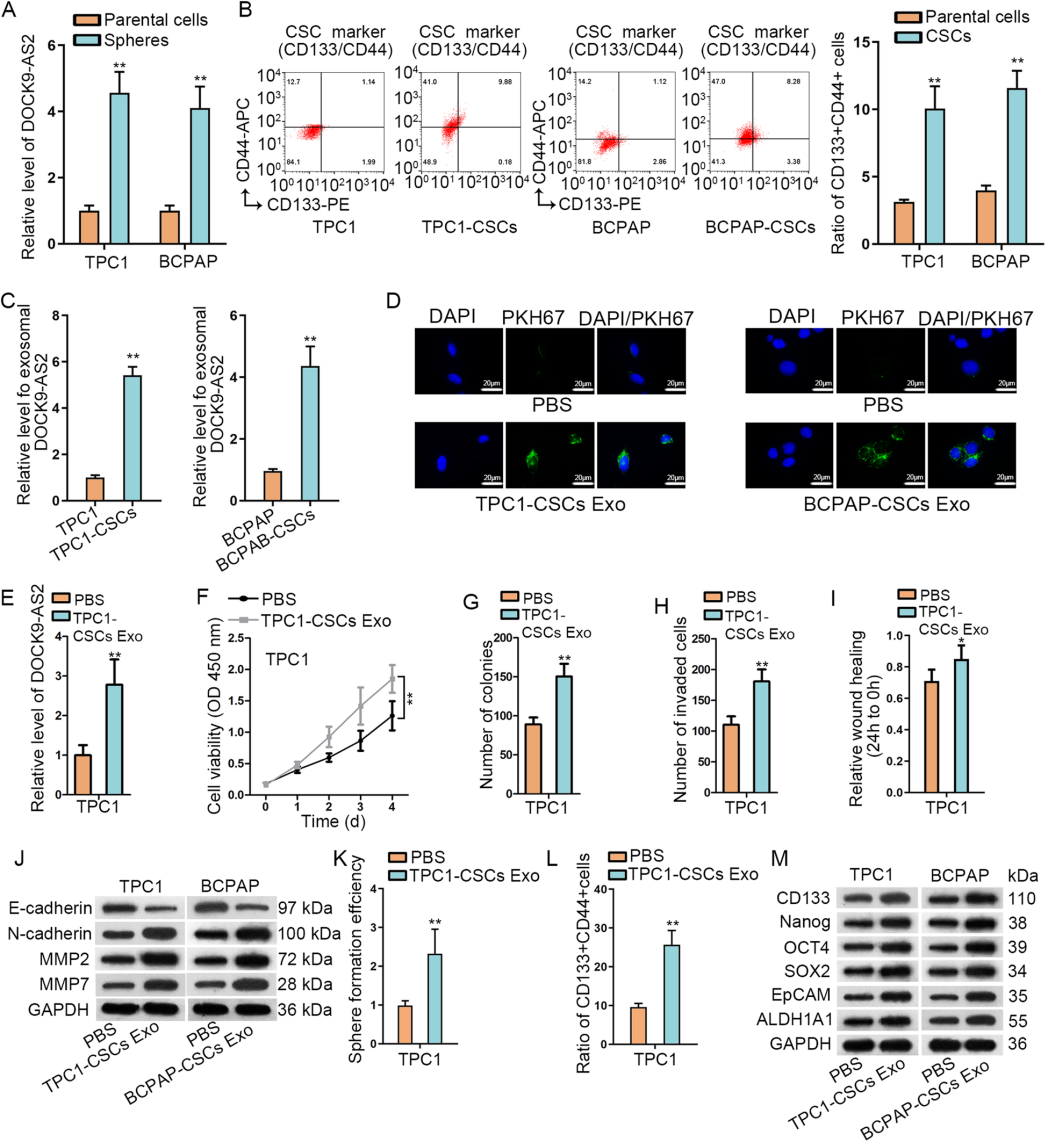
Exosomal DOCK9-AS2 derived from PTC CSCs improved proliferation, migration, invasion, EMT, and stemness of PTC cells. **a** RT-qPCR data of DOCK9-AS2 level in the tumor spheres generated by TPC1 and BCPAP cells versus the parental cells. **b** Flow cytometry plot and quantification of the ratio of CD133+CD44+ in PTC CSCs from the tumor spheres versus parental PTC cells. **c** RT-qPCR data of DOCK9-AS2 level in exosomes from PTC cells and PTC CSCs. **d** Fluorescence picture of PKH67 (the green fluorescence marker) in the cytoplasm of PTC1 and BCPAP cells cultured with exosomes from TPC1-CSCs and BCPAP-CSCs versus PBS control. Scale bar: 10 μm. **e** Level of DOCK9-AS2 in the recipient TPC1 cells cultured with the exosomes of TPC1-CSCs or PBS control. **f** OD value detected by CCK-8 reflected the viability of TPC1 cells treated with TPC1 CSCs-exo versus PBS control. **g**–**i** Quantification of colonies, invaded cells, and wound width in TPC1 cells treated with TPC1 CSCs-exo versus PBS control. **j** Western blot of E-cad, N-cad, MMP2, and MMP7 in TPC1 cells treated with TPC1 CSCs-exo versus PBS control. **k**, **l** Quantification of sphere forming efficiency and CD133+CD44+ cell ratio in TPC1 cells treated with TPC1 CSCs-exo versus PBS control. **m** Western blot of stem cell specific genes in TPC1 cells treated with TPC1 CSCs-exo versus PBS control. ^**^P < 0.01. Error bar denotes Mean ± S.D.

Later, we explored whether exosomal DOCK9-AS2 functioned in the tumor microenvironment. Since DOCK9-AS2 level was higher in the spheres generated by TPC1 and BCPAP cells than the parental cells (Fig. 3a), we then focused on CSCs which have been reported to transmit RNAs to accelerate tumorigenesis and development^30^. In addition, transmission of lncRNAs through exosomes is important for tumor microenvironment communication^28^. Hence, we wondered whether exosomes from PTC-CSCs could transmit DOCK9-AS2 to influence PTC cellular processes. As we observed, DOCK9-AS2 level was not affected under RNase treatment alone, but overtly decreased by co-treatment of RNase and Triton X-100 (Fig. S3A), suggesting that DOCK9-AS2 was packaged by membranes rather than being released. Then, we dissociated PTC-CSCs from tumor spheres derived from TPC1 and BCPAP cells, and recognized these PTC-CSCs with higher CD133^+^CD44^+^ and higher expression of stemness specific proteins than parental cells (Fig. 3b and S3B). Thereafter, we isolated exosomes of PTC-CSCs from the medium, and confirmed the morphology, size and exosomal markers (Fig. S3C). RT-qPCR validated that DOCK9-AS2 level was over 4 fold higher in exosomes of PTC-CSCs than PTC-CSCs (Fig. S3D). Also, DOCK9-AS2 was enriched by approximately 4–5-fold higher in exosomes derived from PTC-CSCs than exosomes from naïve PTC cells (Fig. 3c).

Next, we assessed the communication between PTC-CSCs and PTC cells. Interestingly, we observed the localization of PKH67 in the cytoplasm of TPC1 and BCPAP cells incubated with exosomes from PTC-CSCs (Fig. 3d and S6), which made the recipient TPC1 and BCPAP cells present higher level of DOCK9-AS2 compared to control groups (Fig. 3e and S3E). Thus, we demonstrated that PTC-CSCs transmitted DOCK9-AS2 to recipient PTC cells via exosomes. Furthermore, we validated that exosomes from PTC-CSCs facilitated proliferation, migration, invasion, EMT and stemness of recipient cells (Fig. 3f–m and S3f–k). Collectively, PTC-CSCs delivered exosomal DOCK9-AS2aggravated malignant activities in PTC cells.

### DOCK9-AS2 activated Wnt/β-catenin signaling through transcriptionally inducing CTNNB1

**Fig. 4:**
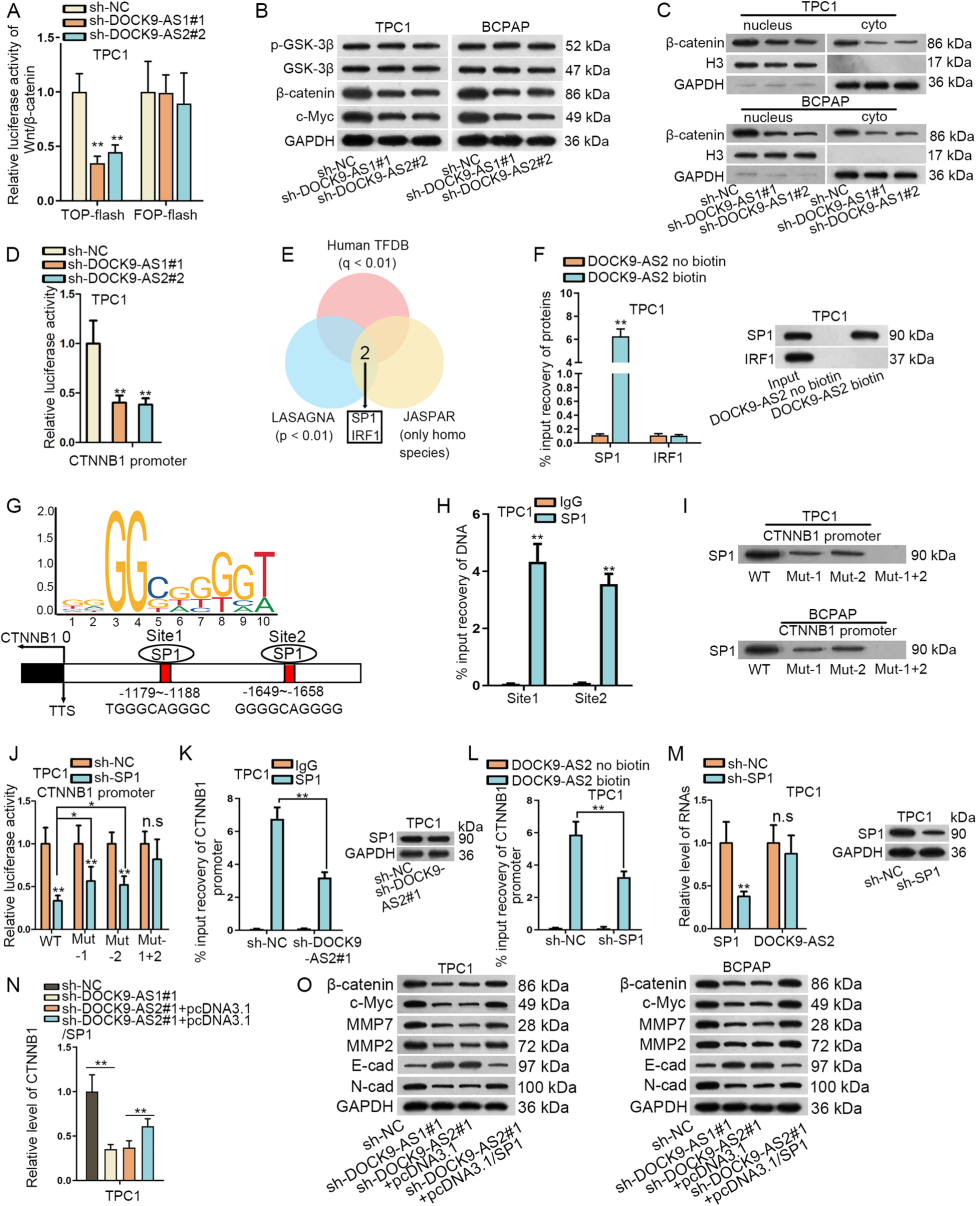
DOCK9-AS2 activated Wnt/β-catenin signaling through transcriptionally inducing CTNNB1. **a** The luciferase activity of Wnt/β-catenin signaling was detected using TOP-flash assay with FOP-flash as negative control. **b** Western blot of p-GSK-3β, GSK-3β, β-catenin, c-Myc in PTC cells under DOCK9-AS2 depletion. **c** The nuclear and cytoplasm levels of β-catenin were examined by western blotting. **d** Luciferase activity of CTNNB1 promoter reporter was tested in PTC cells responding to DOCK9-AS2 depletion. **e** Venn diagram showed SP1 and IRF1 in the intersection of prediction results from Human TFDB, LADAGNA and JASPAR as the potential CTNNB1-targeting TFs. **f** Western blot of the enrichment of SP1 and IRF1 in the pulldown of DOCK9-AS2 biotin group and DOCK9-AS2 non-biotin group. **g** 2 SP1 sites on CTNNB1 promoter was obtained from JASPAR. **h** RT-qPCR tested the enrichment of CTNNB1 promoter fractions with site 1 or site 2 in the ChIP products of SP1. **i** DNA pulldown assay showed the enrichment of SP1 in the pulldown of WT or Mut CTNNB1 promoter. **j** The luciferase activity of CTNNB1 promoter was quantified under SP1 knockdown. **k** RT-qPCR data of the enrichment of CTNNB1 promoter in ChIP products of SP1 in PTC cells with DOCK9-AS2 depletion. **l** RT-qPCR detected the enrichment of CTNNB1 in the pulldown of DOCK9-AS2 biotin group after silencing SP1. **m** RT-qPCR of levels of SP1 mRNA and DOCK9-AS2 and western blot of SP1 protein in PTC cells under SP1 knockdown. **n** RT-qPCR data of CTNNB1 expression in PTC cells transfected with sh-NC, sh-DOCK9-AS1#2, sh-DOCK9-AS2#1+pcDNA3.1 or sh-DOCK9-AS2#1+pcDNA3.1/SP1. **o** Western blot results of β-catenin, c-Myc, MMP7, MMP2, E-cad, and N-cad levels in each group. ^*^P < 0.05, ^**^P < 0.01^.^ n.s. meant no significance. Error bar denotes Mean ± S.D.

Subsequently, we interrogated the regulatory mechanism of DOCK9-AS2 in PTC. Wnt/β-catenin is a renowned pathway that regulates cell proliferation, metastasis, and stemness in cancer^30,31^. Therefore, we wondered whether DOCK9-AS2 influenced Wnt/β-catenin signaling. Expectedly, the TOP-flash activity was reduced with FOP-flash unchanged under DOCK9-AS2 knockdown in TPC1 and BCPAP cells (Fig. 4a and S4A). Besides, DOCK9-AS2 depletion declined the levels of β-catenin in both nucleus and cytoplasm and that of its downstream c-Myc, without any changes on the levels of phosphorylated glycogen synthase kinase 3 beta (p-GSK-3β) and total GSK-3β (Fig. 4b, c). These data indicated that DOCK9-AS2 positively regulated Wnt/β-catenin pathway through regulating β-catenin expression.

LncRNAs regulate target genes through various mechanisms at different levels according to their localization^5,32^. Considering DOCK9-AS2 localized in both nucleus and cytoplasm of PTC cells, we first tested its effect on CTNNB1 (the coding gene of β-catenin) transcription. Unsurprisingly, luciferase activity of CTNNB1 promoter reporter declined in PTC cells responding to DOCK9-AS2 depletion (Fig. 4d and S4B). LncRNAs can interact with transcription factors (TFs) to affect target gene transcription^5,33^. After gathering potential CTNNB1-targeting TFs predicted by Human TFDB (threshold: *q* < 0.01) (http://bioinfo.life.hust.edu.cn/HumanTFDB/#!/), LADAGNA (threshold: *p* < 0.01) (https://biogrid-lasagna.engr.uconn.edu/lasagna_search/) and JASPAR (http://jaspar.genereg.net/), 2 TFs (SP1 and interferon regulatory factor 1 (IRF1)) were confirmed to target CTNNB1 in human (Fig. 4e). Further, pulldown assay showed that only SP1 protein enriched in DOCK9-AS2 biotin group (Fig. 4f and S4C). Hence, we deduced that DOCK9-AS2 recruited SP1 to regulate CTNNB1 transactivation.

Then, JASPAR identified 2 probable SP1 sites on CTNNB1 promoter (Fig. 4g). ChIP analysis confirmed that both CTNNB1 promoter sections with site 1 or 2 were precipitated by SP1 antibody (Fig. 4h and S4D). More importantly, mutation of either site 1 or 2 partly reduced while mutation of both them completely hindered the binding of SP1 to CTNNB1 promoter (Fig. 4i). Besides, SP1 silence reduced the luciferase activity of WT CTNNB1 promoter, and such reduction was partially reversed when mutating site 1 or site 2, whereas that of CTNNB1 promoter with co-mutation of site 1 and 2 remained unchanged (Fig. 4j and S4e). These data indicated that SP1 bound to CTNNB1 promoter at both site 1 and 2.

Later, we examined whether DOCK9-AS2 cooperated with SP1 to regulate CTNNB1 transcription. We discovered that silencing DOCK9-AS2 reduced SP1-binding with CTNNB1 promoter, but did not affect SP1 protein expression (Fig. 4k and S4F). Meanwhile, SP1 silence hindered the interaction between CTNNB1 promoter and DOCK9-AS2 (Fig. 4l and S4G), without any influences on DOCK9-AS2 expression (Fig. 4m and S4H). Then, we found that overexpressing SP1 only partially restored DOCK9-AS2 silence-lowered CTNNB1 expression (Fig. 4n and S4I). Furthermore, the impact of DOCK9-AS2 deficiency on the levels of β-catenin, c-Myc, MMP7, MMP2, and N-cad, and E-cad, were partly counteracted by SP1 overexpression (Fig. 4o). Altogether, DOCK9-AS2 regulated CTNNB1 expression and Wnt/β-catenin pathway partly through SP1-mediated CTNNB1 transactivation.

### DOCK9-AS2 upregulated CTNNB1 and activated Wnt/β-catenin pathway through sponging miR-1972

**Fig. 5:**
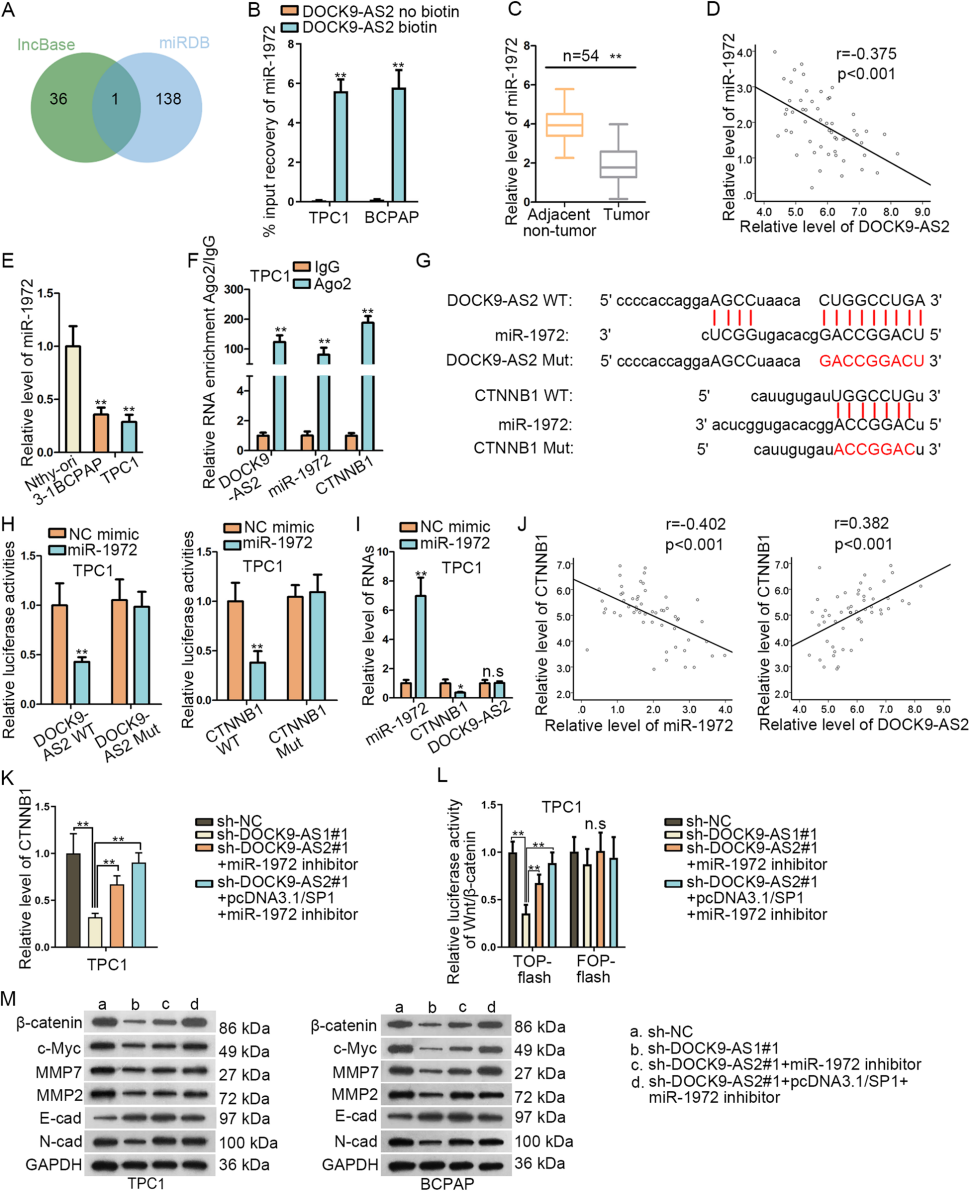
DOCK9-AS2 upregulated CTNNB1 and activated Wnt/β-catenin pathway through sponging miR-1972. **a** The miRNAs potentially binding to DOCK9-AS2 and CTNNB1 were predicted through lncBase and miRDB. **b** RT-qPCR of miR-1972 enrichment in the pulldown of DOCK9-AS2 biotin probe and non-biotin probe groups. **c** RT-qPCR of miR-1972 level in PTC tissues versus adjacent normal ones. **d** Correlation of miR-1972 and DOCK9-AS2 in PTC samples was assayed by Pearson’s *χ*2 analysis. **e** RT-qPCR analysis of miR-1972 level in PTC cell lines. **f** RT-qPCR analysis of the miR-1972, DOCK9-AS2 and CTNNB1 enrichment in RIP precipitates of anti-Ago2 in PTC cells. **g** The predicted miR-1972 binding sites in DOCK9-AS2 and CTNNB1 and designed mutated sites. **h** Luciferase activities of DOCK9-AS2 WT/Mut and CTNNB1 WT/Mut in PTC cells treated with miR-1972 mimic or NC mimic. **i**. RT-qPCR data of levels of miR-1972, DOCK9-AS2 and CTNNB1 in PTC cells transfected with miR-1972 mimic or NC mimic. **j** Pearson’s correlation analysis of the correlations of CTNNB1 with miR-1972 and DOCK9-AS2 in PTC tissues. **k** RT-qPCR data of CTNNB1 expression in PTC cells transfected with sh-NC, sh-DOCK9-AS1#1, sh-DOCK9-AS2#1+miR-1972 inhibitor or sh-DOCK9-AS2#1+pcDNA3.1/SP1+miR-1972 inhibitor. **l** TOP-flash assay tested the Wnt/β-catenin activity in PTC cells with indicated transfections. **m** Western blot analysis for β-catenin, c-Myc, MMP7, MMP2, E-cad, N-cad levels in each group. ^*^P < 0.05, ^**^P < 0.01^.^ n.s. meant no significance. Error bar denotes Mean ± S.D.

Thereafter, we explored other possible manner for DOCK9-AS2 to affect CTNNB1 expression. Considering that DOCK9-AS2 was also located in cytoplasm, we deduced that DOCK9-AS2 regulated CTNNB1 post-transcriptionally. It is well-established that lncRNAs function through ceRNA network at post-transcriptional level^34,35^. Thus, we predicted the miRNAs shared by DOCK9-AS2 and CTNNB1 through lncBase (http://carolina.imis.athena-innovation.gr/diana_tools/web/index.php?r=lncbasev2%2Findex-predicted) and miRDB (http://mirdb.org/index.html). As a result, only miR-1972 was identified by both tools (Fig. 5a). Besides, miR-1972 was pulled down by DOCK9-AS2 biotin probe (Fig. 5b). Further, we confirmed that miR-1972 was downregulated and negatively correlated with DOCK9-AS2 in PTC samples (Fig. 5c, d). Also, we confirmed miR-1972 downregulation in PTC cell lines (Fig. 5e). Then, RIP analysis showed that miR-1972, DOCK9-AS2 and CTNNB1 were all precipitated by anti-Ago2 in PTC cells (Fig. 5f and S5A). After that, we identified miR-1972 sites in DOCK9-AS2 and CTNNB1, and also designed the mutated sequences (Fig. 5g). As anticipated, upregulated miR-1972 reduced the luciferase activities of DOCK9-AS2 WT and CTNNB1 WT, whereas no impacts on that of DOCK9-AS2 Mut and CTNNB1 Mut (Fig. 5h and S5B). Besides, we revealed that overexpressing miR-1972 reduced CTNNB1 level but failed to influence DOCK9-AS2 expression in PTC cells (Fig. 5i and S5C). In addition, CTNNB1 was proved to be negatively related to miR-1972 and positively related to DOCK9-AS2 level in PTC samples (Fig. 5j). More importantly, the impacts of DOCK9-AS2 depletion on CTNNB1 expression and Wnt/β-catenin activity were partially restored by inhibiting miR-1972, but fully reversed when transfecting miR-1972 inhibitor+pcDNA3.1/SP1 (Fig. 5k, l and S5D, E). Also, similar phenomena were observed on the levels of several proteins affected by DOCK9-AS2 (Fig. 5m). In a word, DOCK9-AS2 affected CTNNB1 and Wnt/β-catenin pathway through targeting both miR-1972 and SP1.

### DOCK9-AS2 regulated proliferation, migration, invasion, EMT and stemness in PTC cells through Wnt/β-catenin pathway

**Fig. 6:**
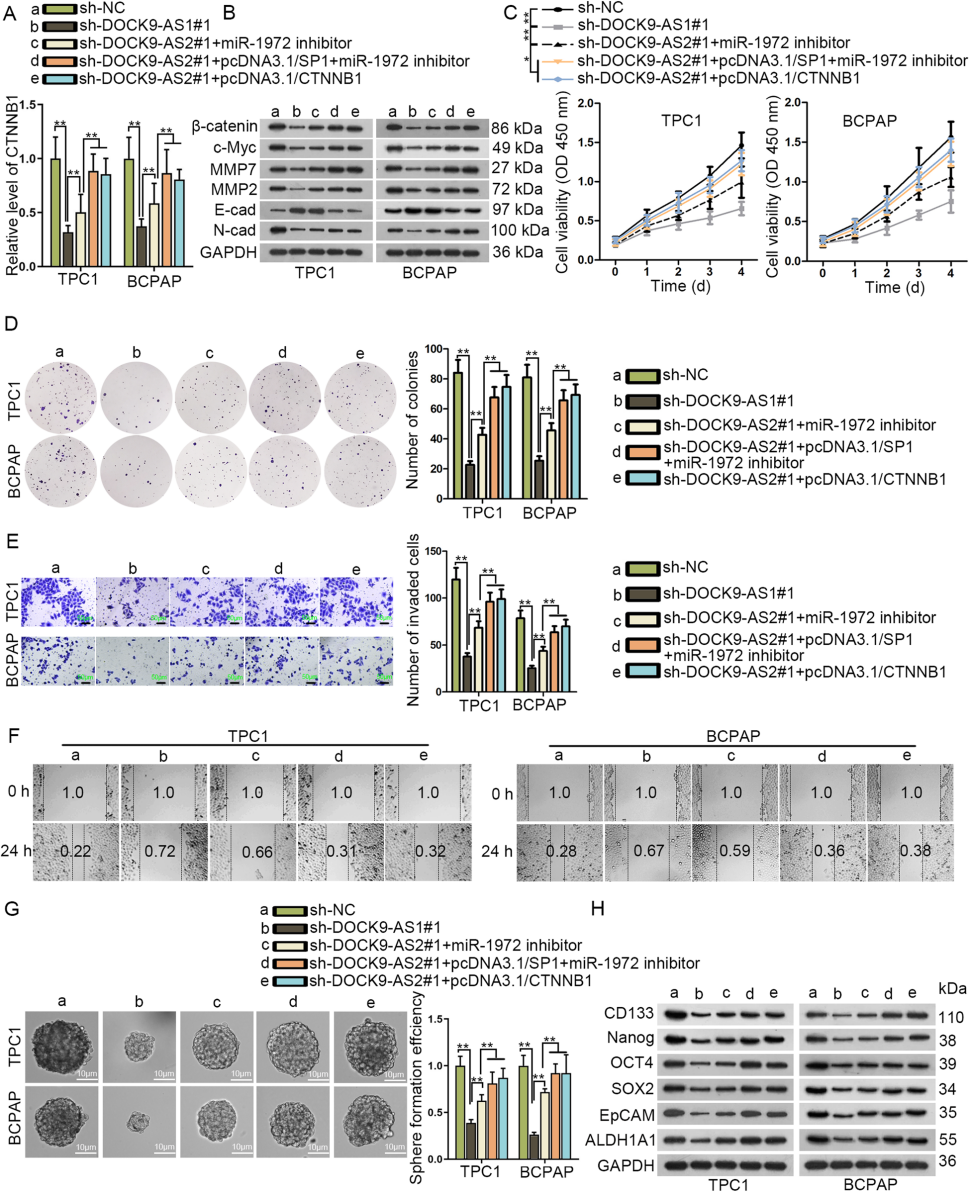
DOCK9-AS2 regulated proliferation, migration, invasion, EMT and stemness in PTC cells through Wnt/β-catenin pathway. **a** CTNNB1 expression was evaluated by RT-qPCR in PTC cells transfected with sh-NC, sh-DOCK9-AS1#1, sh-DOCK9-AS2#1+miR-1972 inhibitor, sh-DOCK9-AS2#1+pcDNA3.1/SP1+miR-1972 inhibitor or sh-DOCK9-AS2#1+pcDNA3.1/CTNNB1. **b** Western blots of the downstream effectors (c-Myc, MMP2, MMP7, E-cad, and N-cad) in PTC cells of each group. **c** CCK-8 evaluated the viable PTC cells with indicated transfections. **d** Pictures and quantification of colonies generated by PTC cells with indicated transfections. **e** Pictures of invaded PTC cells under indicated transfections were taken and number of invasive cells were quantified in each field. Scale bar: 100 μm. **f** Pictures of scratch wound in PTC cells with wound width labeled. **g** Pictures and quantification of 3rd generation spheres formed by PTC cells with indicated transfection. Scale bar: 50 μm. **h** Western blot of stem cell specific genes in PTC cells of each group. ^*^P < 0.05, ^**^P < 0.01 n.s. meant no significance. Error bar denotes Mean ± S.D.

Subsequently, we aimed to verify the effect of DOCK9-AS2/SP1/miR-1972/CTNNB1 axis in PTC. Consistent with changes on CTNNB1 expression, we confirmed that β-catenin level and its downstream effectors in DOCK9-AS2-silenced PTC cells were partially restored by inhibiting miR-1972, but fully normalized with transfection of miR-1972 inhibitor+pcDNA3.1/SP1 or pcDNA3.1/CTNNB1 (Fig. 6a, b). Functionally, miR-1972 inhibition partially recovered proliferation, migration, invasion, and stemness in TPC1 and BCPAP cells with DOCK9-AS2 knockdown, and further upregulated SP1 deepened such restoration. Meanwhile, CTNNB1 overexpression completely recovered the effect of DOCK9-AS2 knockdown on above behaviors (Fig. 6c–h). Therefore, it was suggested that DOCK9-AS2 regulated malignant phenotypes in PTC cells through SP1 and miR-1972-regulated CTNNB1.

### Exosomal DOCK9-AS2 derived from PTC-CSCs induced tumorigenesis and metastasis of PTC in vivo

**Fig. 7:**
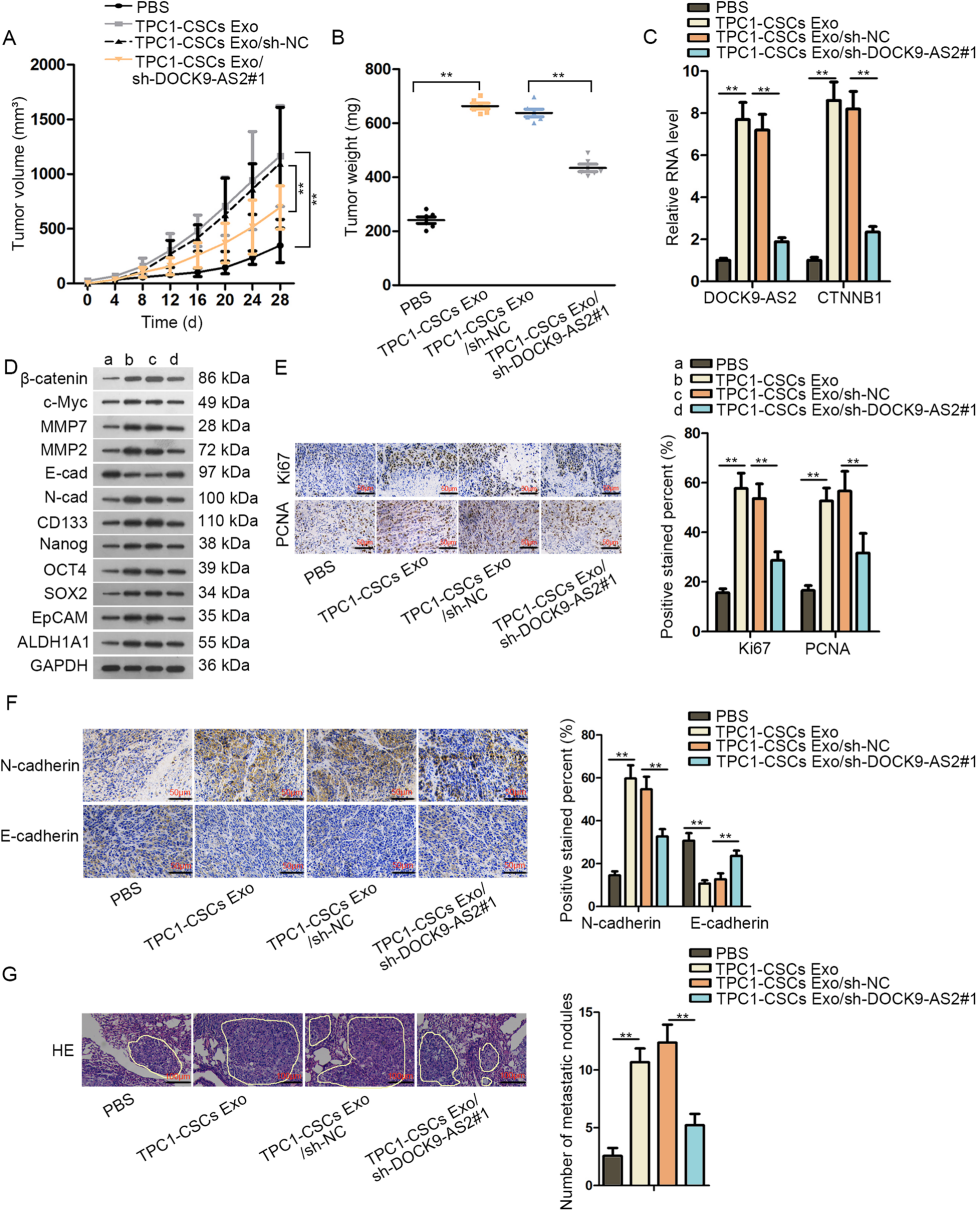
Exosomal DOCK9-AS2 derived from PTC CSCs induced tumorigenesis and metastasis of PTC in vivo. The exosomes from TPC1-CSCs transfected with sh-NC or sh-DOCK9-AS2#1 were extracted and incubated with naïve TPC1 cells. **a** Transfected TPC1 cells were subcutaneously inoculated into mice. Tumor growth volume was detected 4 days a time until day 28 to generate growth curve. **b** Tumor weight was tested at day 28 after sacrificing the mice in each group. **c** RT-qPCR data of DOCK9-AS2 and CTNNB1 expression levels in xenografts of each group. **d** Western blotting for β-catenin, c-Myc, MMP7, MMP2, N-cad, E-cad, CD133, Nanog, OCT4, and SOX2 in tumors from mice of each group. **e**, **f** The IHC pictures and quantification of stain positivity of Ki67, PCNA, E-cad, and N-cad in mice of each group. Scale bar: 100 μm. **g** Transfected TPC1 cells were intravenously injected into mice to form lung metastasis. Pictures of HE staining for the metastatic nodules in each group were taken and the nodules of the lung in mice were counted manually. Scale bar: 100 μm. ^**^P < 0.01. Error bar denotes Mean ± S.D.

Finally, we evaluated the effect of exosomal DOCK9-AS2 derived from PTC-CSCs on PTC in vivo. Results showed that exosomes-facilitated tumor growth was blocked after DOCK9-AS2 silence in TPC1-CSCs (Fig. 7a, b). Likewise, exosomes from TPC1-CSCs induced DOCK9-AS2 and CTNNB1 expressions in xenografts, and silencing DOCK9-AS2 in these CSCs reversed such induction (Fig. 7c). Also, similar effects were observed in the levels of proteins related to Wnt pathway, EMT and stemness, as well as the staining of growth-(Ki67 and PCNA) and EMT-associated markers (E-cadherin and N-cadherin), in above tumors(Fig. 7d–f). Moreover, the metastatic nodules were increased in TPC1-CSCs Exo group, and such increasement were reversed under DOCK9-AS2 inhibition in TPC1-CSCs (Fig. 7g). In conclusion, exosomal DOCK9-AS2 derived from PTC-CSCs induced tumorigenesis and metastasis of PTC in vivo

## Discussion

The functional role of exosomal lncRNAs has been reported in cancer development, including in PTC^36^. Herein, we first uncovered the correlation between DOCK9-AS2 and PTC by identifying its upregulation in PTC tissues and cell lines. Notably, we discovered the existence of DOCK9-AS2in exosomes of PTC patients and cell lines. Besides, we unveiled the tumor-contributing effect of DOCK9-AS2 on PTC cell proliferation, motility, EMT, and stemness, indicating a novel thought to hinder PTC progression.

Former studies have illustrated that lncRNAs promoted PTC development and by regulating stemness^37–39^. CSCs are identified as crucial participants in tumor progression^16,17^. Importantly, considering the highly dynamic equilibrium state of CSCs and non-CSCs^18,19^, non-CSCs could dedifferentiate into CSCs^20^. Increasing reports suggested the function of exosomes in transmitting molecules between cells, especially between non-CSCs and CSCs^40,41^. Presently, we validated that exosomes from PTC-CSCs increased DOCK9-AS2 level and facilitated the progression in PTC both in vitro and in vivo. These findings suggested that DOCK9-AS2 might influence the exosome-mediated cross-talk between non-CSCs and CSCs in PTC to drive PTC progression.

Wnt/β-catenin pathway is a key pathway responsible for cancer development, PTC included^42,43^. It is known that activation of Wnt/β-catenin pathway depends on nuclear translocation of β-catenin^44^, and GSK-3β is a regular inhibitor of β-catenin phosphorylation^45^. Our data first supported that DOCK9-AS2 positively regulated Wnt/β-catenin activity by increasing β-catenin level but did not influence GSK-3β and p-GSK-3β.

Intriguingly, here we disclosed that DOCK9-AS2 located in both nucleus and cytoplasm of PTC cells. In nucleus, lncRNAs can interact with TFs to modulate transcription of target genes^5^. Consistently, SP1, a well-known oncogenic TF in cancers^46,47^ was identified as the TF targeting CTNNB1 in PTC. Previously, the relation of SP1 to Wnt/β-catenin pathway has already been suggested^48,49^, but the direct interaction between SP1 and CTNNB1 was first revealed by our study. More significantly, we unmasked that DOCK9-AS2 activated CTNNB1 transcription by cooperating rather than regulating SP1. Interestingly, we also found that cytoplasmic DOCK9-AS2 regulated CTNNB1 through acting as a ceRNA by sequestering miR-1972. MiR-1972 is found as a tumor-suppressor in osteosarcoma^50^. Moreover, miR-1972 inhibition plus SP1 overexpression fully rescued Wnt/β-catenin signaling activity in DOCK9-AS2-silenced PTC cells, therefore recovering cellular progression. These findings indicated that DOCK9-AS2 activated Wnt/β-catenin pathway through SP1 and miR-1972-regulated CTNNB1, resulting in accelerated PTC progression.

## Conclusions

**Fig. 8:**
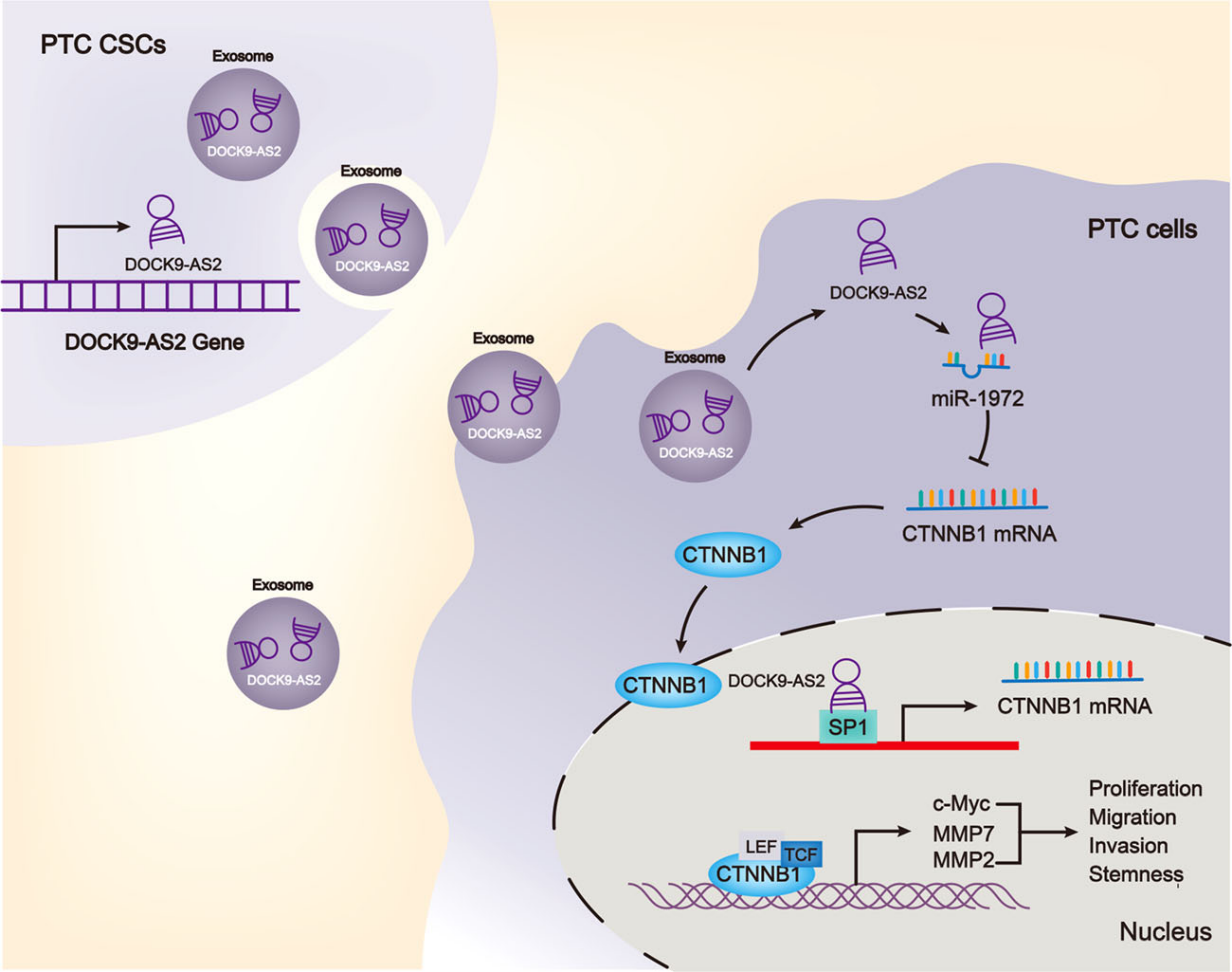
Graphical abstract. Exosomal lncRNA DOCK9-AS2 derived from PTC-CSCs induced CTNNB1 transcription by interacting with SP1 and sponged miR-1972 to induce CTNNB1 level to activate Wnt/β-catenin and PTC proliferation, migration, invasion, and stemness.

This study first revealed that DOCK9-AS2 was an exosomal lncRNA derived from PTC-CSCs and upregulated in PTC. Functionally, PTC-CSCs transferred DOCK9-AS2 to naïve PTC cells to facilitate cellular progression in PTC. Mechanistically, DOCK9-AS2 increased CTNNB1 expression by SP1 and miR-1972 to activate Wnt/β-catenin pathway (Fig. 8). These findings indicated that DOCK9-AS2 was a promising target for PTC therapy. However, this study is limited because of the small number of clinical samples. Therefore, we will dedicate ourselves to enlarge the clinical significance of DOCK9-AS2 in PTC.

## Supplementary information

Supplementary figure legends

Figure S1

Figure S2

Figure S3

Figure S4

Figure S5

Figure S6
