# Supplementary material for: Exosomal lncRNA DOCK9-AS2 derived from cancer stem cell-like cells activated Wnt/β-catenin pathway to aggravate stemness, proliferation, migration, and invasion in papillary thyroid carcinoma
Source: Cell Death Dis. 2020 Sep 11;11(9):743. doi: 10.1038/s41419-020-02827-w (PMC7486896; doi:10.1038/s41419-020-02827-w)
Supplement: Supplementary file 1 — Supplementary figure legends [file 41419_2020_2827_MOESM1_ESM.docx]

**Figure S1 A.** DOCK9-AS2 expression level in THCA specimens versus normal specimens from TCGA data analysis in GEPIA. **B-C.** Images of the morphology and size of isolated exosomes through TEM and NTA analysis. The western blotting for the exosome markers CD81, CD63, TSG-101, Alix, and Calnexin (negative control) in the exosomes of PTC cases (n = 54) and healthy control (n = 44) and in TPC1, BCPAB and Nthy-ori3-1 cells. **D.** RT-qPCR data of DOCK9-AS2 level in exosomes from PTC cells and normal cells versus the producer PTC cells and normal cells. **E.** Data of DOCK9-AS2 in Coding Potential calculator (CPC) bioinformatics tool. ^*^P < 0.05, ^**^P < 0.01. n.s. meant no significance. Error bar denotes Mean ± S.D.

**Figure S2 A.** RT-qPCR confirmed the silence of DOCK9-AS2 expression by two DOCK9-AS2 specific shRNAs in TPC1 and BCPAB cells. **B-C.** Proliferation of TPC1 and BCPAB cells were assessed by CCK-8 and colony formation assays after transfection with sh-DOCK9-AS2#1/2. **D.** Transwell assay was performed for detecting invasive BCPAB cells responding to DOCK9-AS2 depletion. Scale bar: 100μm. **E.** Wound-healing assay for the migration analysis of BCPAB cells in response to DOCK9-AS2 knockdown. **F.** The IF staining image for the fluorescence intensity of E-cadherin and N-cadherin. Scale Bar: 10μm. **G.** Sphere-forming assay for the effect of DOCK9-AS2 silence on tumor spheres. Scale bar: 100μm. **H.** Ratio of CD44+CD133+ BCPAB cells were measured by flow cytometry method. **I.** The SP cells were sorted from BCPAB cells with DOCK9-AS2 knockdown by flow cytometry. ^**^P < 0.01. Error bar denotes Mean ± S.D.

**Figure S3 A.** RT-qPCR data of DOCK9-AS2 level in the medium of TPC1 and BAPAB cells treated with RNase A or RNase A + Triton x-100. **B.** Western blot of stem markers in PTC-CSCs and naïve PTC cells. **C.** The morphology, size and exosomal markers were confirmed in exosomes of PTC CSCs and PTC cells by TEM, NTA, and western blot. **D.** Level of DOCK9-AS2 in the recipient BCPAB cells after mixing with the exosomes of BCPAB-CSCs. **E.** Level of DOCK9-AS2 in the recipient BCPAB cells after mixing with the exosomes of BCPAB-CSCs. **F.** OD value detected by CCK-8 reflected the viability of BCPAB cells treated with BCPAB CSCs-exo versus PBS control. **G-I.** Quantification of colonies, invaded cells, and wound width in BCPAB cells treated with BCPAB CSCs-exo versus PBS control. **J-K.** Quantification of sphere forming efficiency and CD133+CD44+ cell ratio in BCPAB cells treated with TPC1 CSCs-exo versus PBS control. ^**^P < 0.01. Error bar denotes Mean ± S.D.

**Figure S4 A.** The luciferase activity of Wnt/β-catenin signaling was detected using TOP-flash assay with FOP-flash as negative control. **B.** Luciferase activity of CTNNB1 promoter reporter was tested in PTC cells responding to DOCK9-AS2 depletion. **C.** Western blot of the enrichment of SP1 and IRF1 in the pulldown of DOCK9-AS2 biotin group and DOCK9-AS2 non-biotin group. **D.** RT-qPCR tested the enrichment of CTNNB1 promoter fractions with site 1 or site 2 in the ChIP products of SP1. **E.** The luciferase activity of CTNNB1 promoter was quantified under SP1 knockdown. **F.** RT-qPCR data of the enrichment of CTNNB1 promoter in ChIP products of SP1 in PTC cells with DOCK9-AS2 depletion. **G.** RT-qPCR detected the enrichment of CTNNB1 in the pulldown of DOCK9-AS2 biotin group after silencing SP1. **H.** RT-qPCR of levels of SP1 mRNA and DOCK9-AS2 and western blot of SP1 protein in PTC cells under SP1 knockdown. **I.** RT-qPCR data of CTNNB1 expression in PTC cells transfected with sh-NC, sh-DOCK9-AS1#2, sh-DOCK9-AS2#1 + pcDNA3.1 or sh-DOCK9-AS2#1 + pcDNA3.1/SP1. ^*^P < 0.05, ^**^P < 0.01. n.s. meant no significance. Error bar denotes Mean ± S.D.

**Figure S5 A.** RT-qPCR analysis of the miR-1972, DOCK9-AS2 and CTNNB1 enrichment in RIP precipitates of anti-Ago2 in PTC cells. **B.** Luciferase activities of DOCK9-AS2 WT/Mut and CTNNB1 WT/Mut in PTC cells treated with miR-1972 mimic or NC mimic. **C.** RT-qPCR data of levels of miR-1972, DOCK9-AS2 and CTNNB1 in PTC cells transfected with miR-1972 mimic or NC mimic. **D.** RT-qPCR data of CTNNB1 expression in PTC cells transfected with sh-NC, sh-DOCK9-AS1#1, sh-DOCK9-AS2#1 + miR-1972 inhibitor or sh-DOCK9-AS2#1 + pcDNA3.1/SP1 + miR-1972 inhibitor. **E.** TOP-flash assay tested the Wnt/β-catenin activity in PTC cells with indicated transfections. ^*^P < 0.05, ^**^P < 0.01. n.s. meant no significance. Error bar denotes Mean ± S.D.

**Figure S6** The localization of PKH67 (the green fluorescence marker) in the cytoplasm of PTC1 and BCPAB cells cultured with exosomes from TPC1-CSCs and BCPAB-CSCs vs. PBS control. Scale bar: 10μm.
