# Supplementary figures and images for: Exosomal lncRNA DOCK9-AS2 derived from cancer stem cell-like cells activated Wnt/β-catenin pathway to aggravate stemness, proliferation, migration, and invasion in papillary thyroid carcinoma
Source: Cell Death Dis. 2020 Sep 11;11(9):743. doi: 10.1038/s41419-020-02827-w (PMC7486896; doi:10.1038/s41419-020-02827-w)

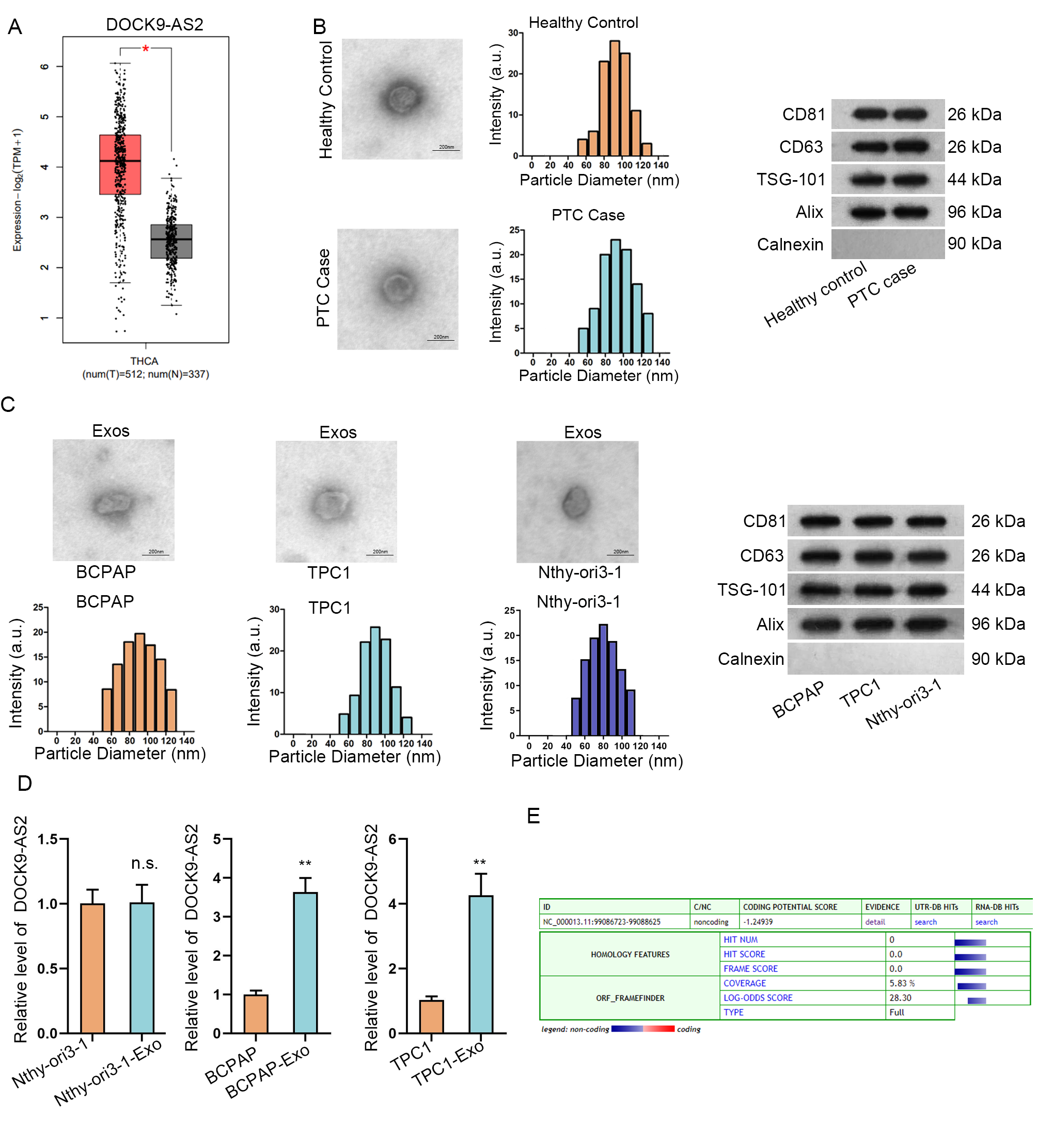

Supplement: Supplementary file 2 — Figure S1 [file 41419_2020_2827_MOESM2_ESM.tif]

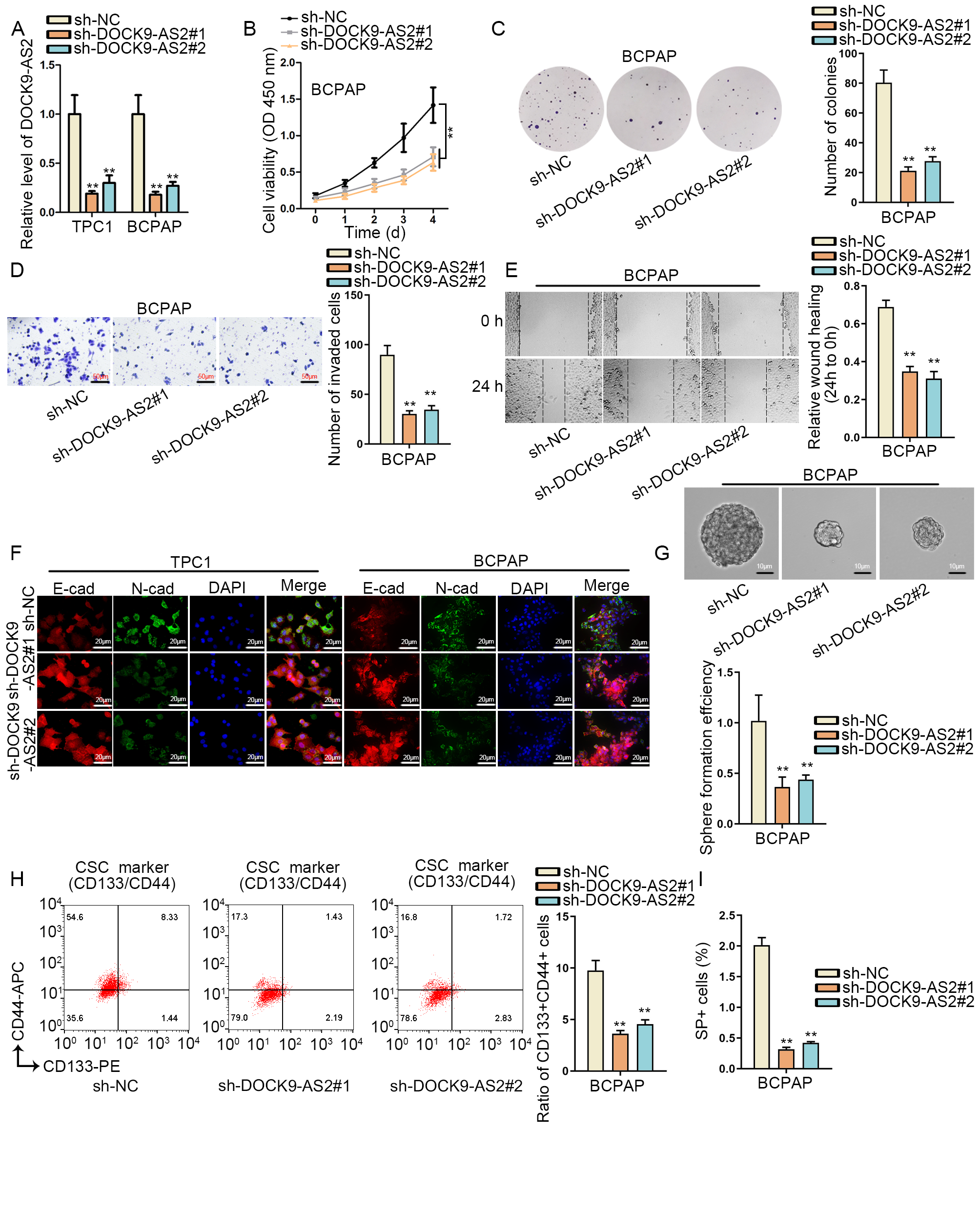

Supplement: Supplementary file 3 — Figure S2 [file 41419_2020_2827_MOESM3_ESM.tif]

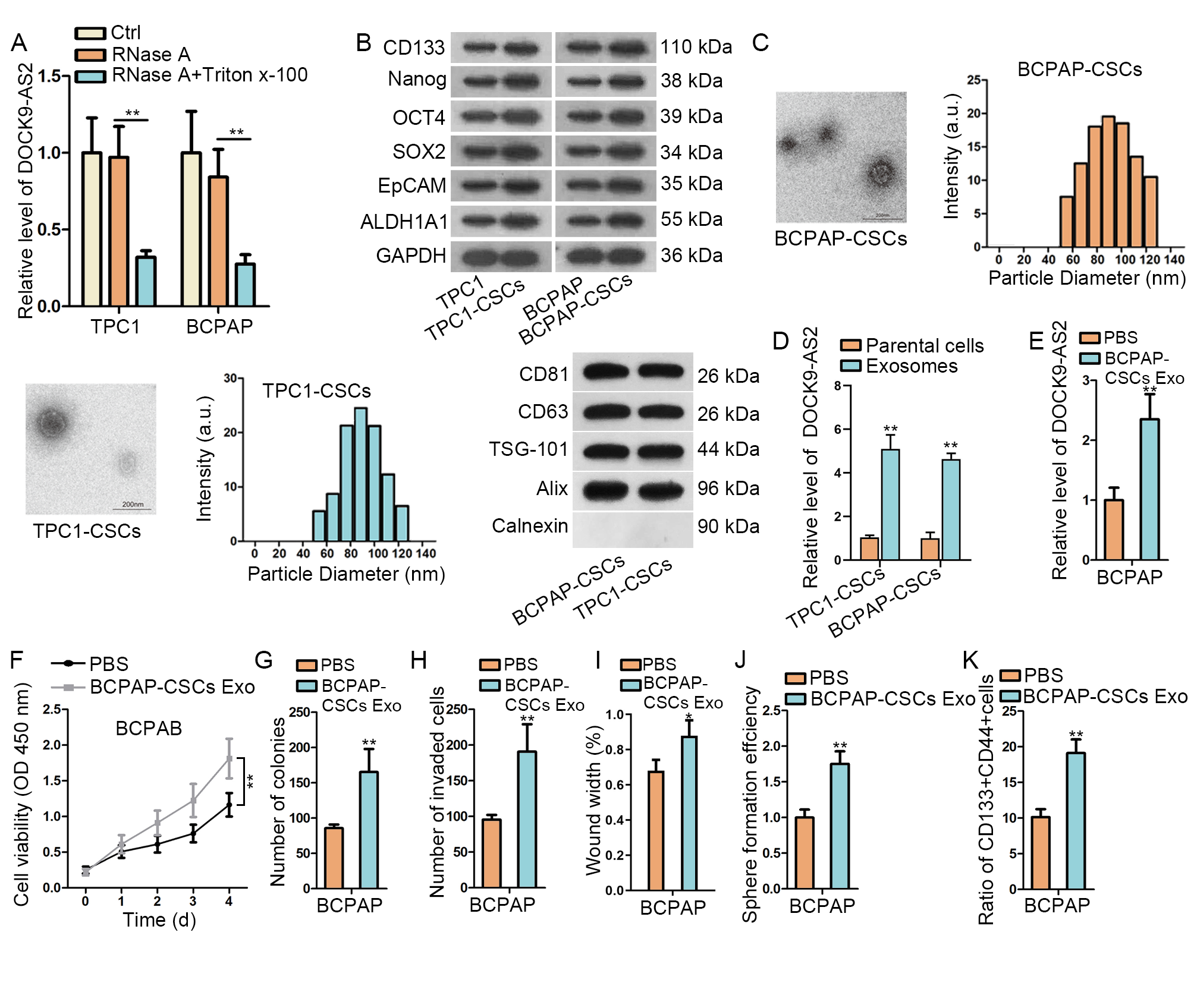

Supplement: Supplementary file 4 — Figure S3 [file 41419_2020_2827_MOESM4_ESM.tif]

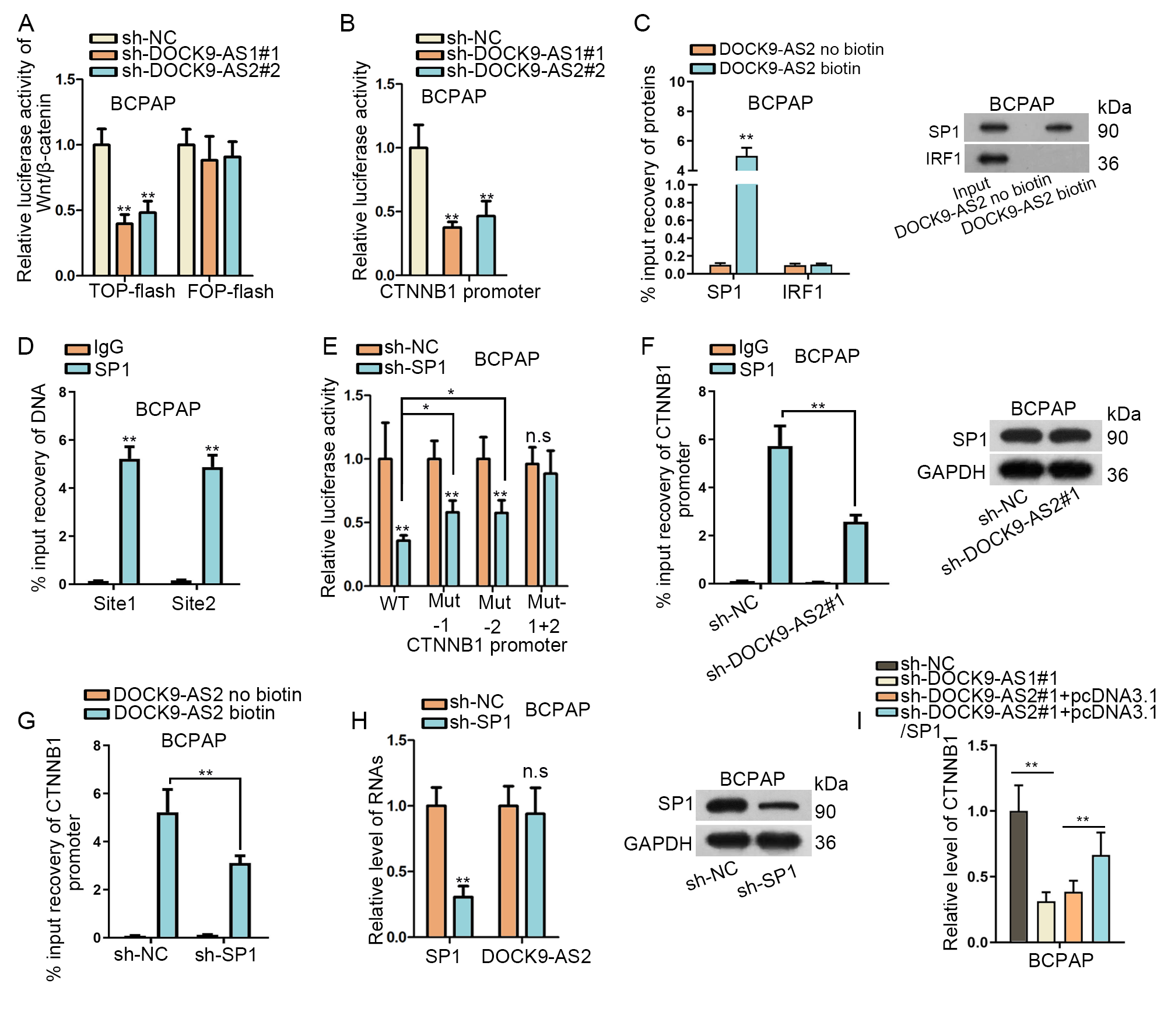

Supplement: Supplementary file 5 — Figure S4 [file 41419_2020_2827_MOESM5_ESM.tif]

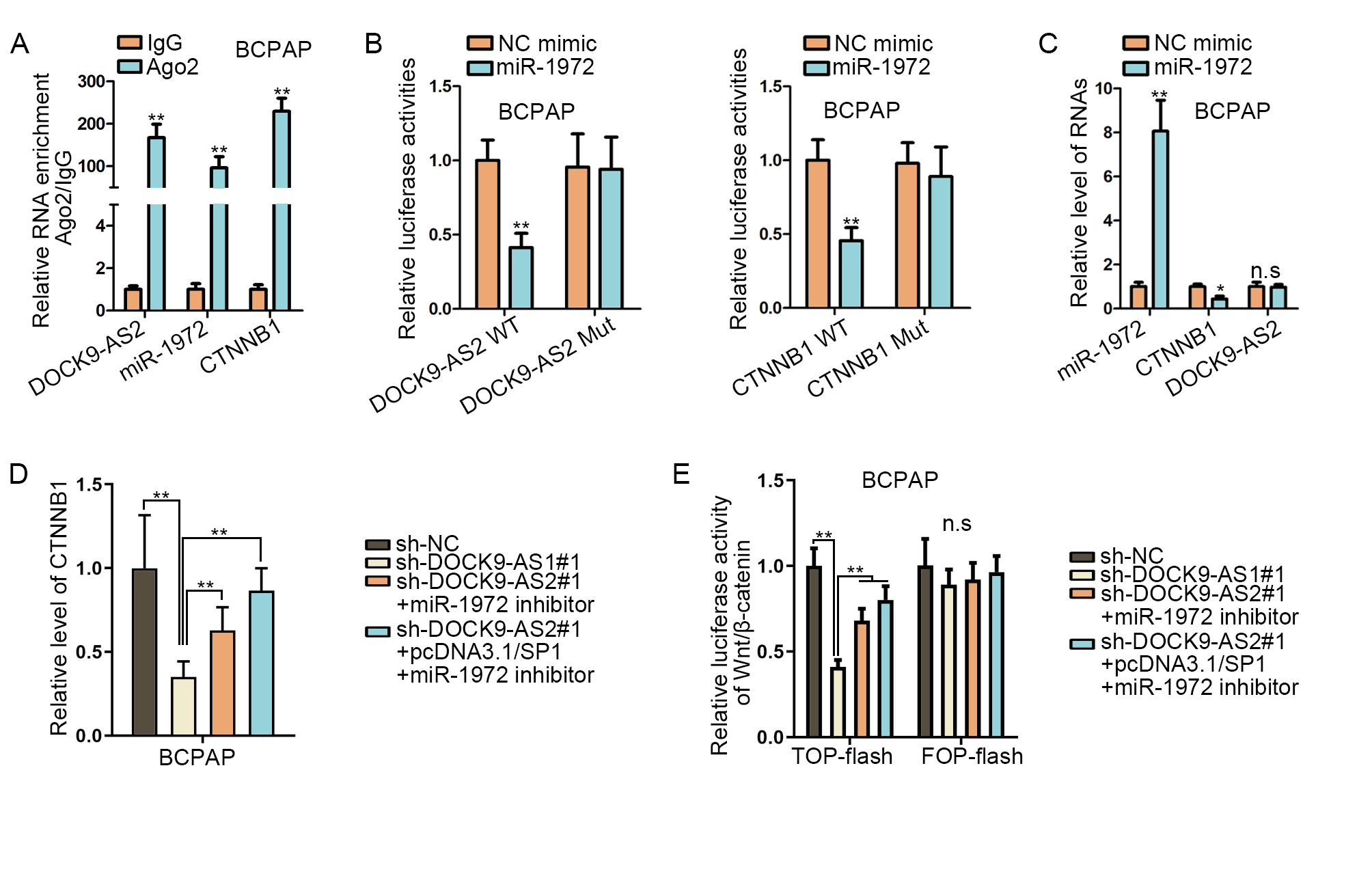

Supplement: Supplementary file 6 — Figure S5 [file 41419_2020_2827_MOESM6_ESM.tif]

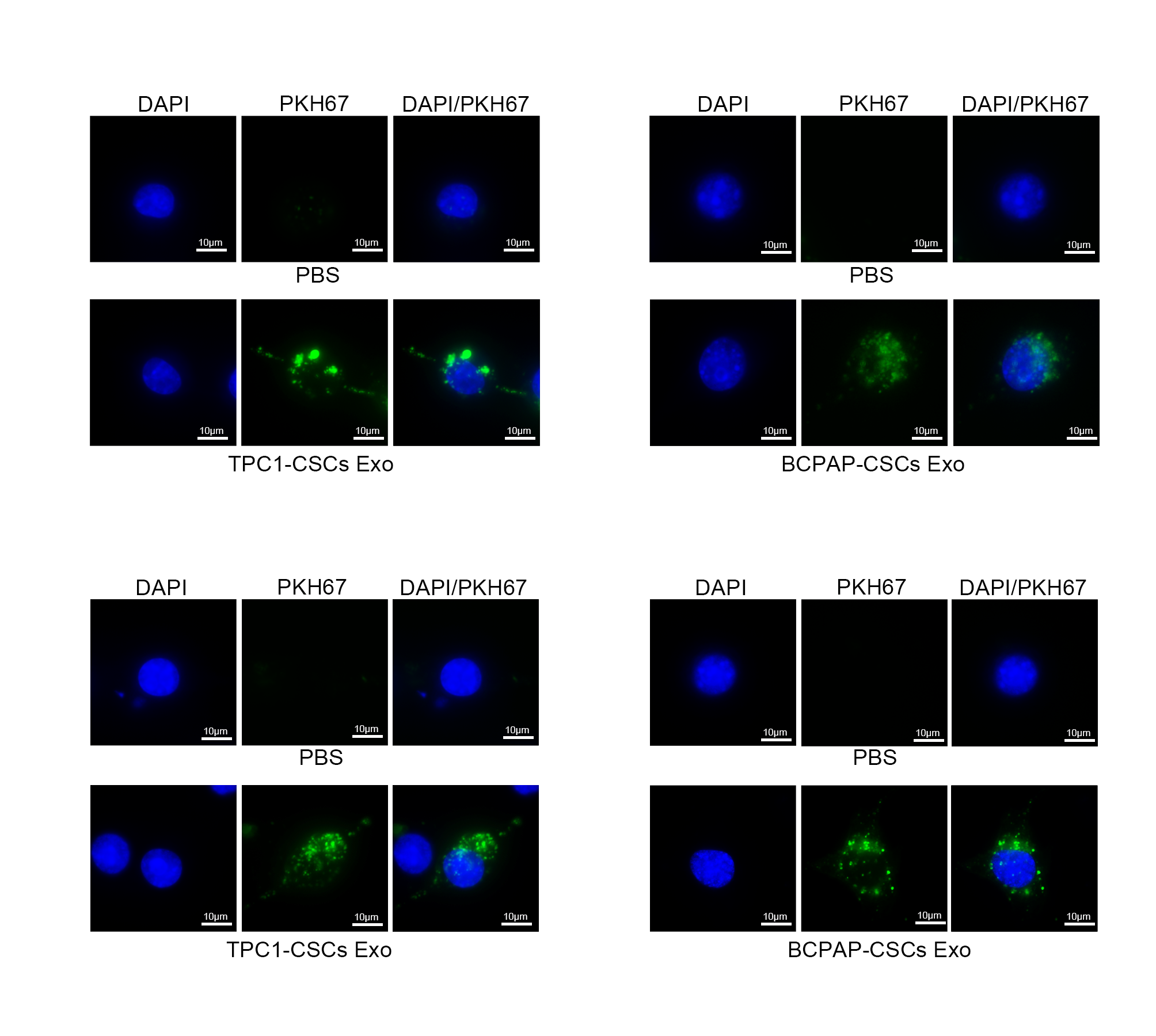

Supplement: Supplementary file 7 — Figure S6 [file 41419_2020_2827_MOESM7_ESM.tif]
